# VEGFR-Mediated Cytotoxic Activity of *Pulicaria undulata* Isolated Metabolites: A Biological Evaluation and In Silico Study

**DOI:** 10.3390/life11080759

**Published:** 2021-07-28

**Authors:** Sameh S. Elhady, Reda F. A. Abdelhameed, Salwa H. Zekry, Amany K. Ibrahim, Eman S. Habib, Khaled M. Darwish, Reem M. Hazem, Khadijah A. Mohammad, Hashim A. Hassanean, Safwat A. Ahmed

**Affiliations:** 1 Department of Natural Products, Faculty of Pharmacy, King Abdulaziz University, Jeddah 21589, Saudi Arabia; ssahmed@kau.edu.sa; 2 Department of Pharmacognosy, Faculty of Pharmacy, Suez Canal University, Ismailia 41522, Egypt; omarreda_70@yahoo.com (R.F.A.A.); solayossif@gmail.com (S.H.Z.); am_kamal66@yahoo.com (A.K.I.); emy_197@hotmail.com (E.S.H.); hashem_omar@pharm.suez.edu.eg (H.A.H.); 3 Department of Pharmacognosy, Faculty of Pharmacy, Sinai University, El-Arish 45511, Egypt; 4 Department of Medicinal Chemistry, Faculty of Pharmacy, Suez Canal University, Ismailia 41522, Egypt; khaled_darwish@pharm.suez.edu.eg; 5 Department of Pharmacology and Toxicology, Faculty of Pharmacy, Suez Canal University, Ismailia 41522, Egypt; reem_ahmed@pharm.suez.edu.eg; 6 Department of Pharmaceutical Chemistry, Faculty of Pharmacy, King Abdulaziz University, Jeddah 21589, Saudi Arabia; kmohammad@kau.edu.sa

**Keywords:** *Pulicaria undulata*, flavonoids, cytotoxic activity, Ehrlich’s ascites carcinoma, VEGF modulator, angiogenesis, human estrogen nuclear receptor-alpha (*h*ER-α), molecular docking

## Abstract

Natural products play a remarkable role not only in the synthesis, design, and discovery of new drugs but also as the most prominent source of drugs and bioactive substances. Adding to the search for new sources of safe innovative antitumor drugs, here we reported a phytochemical study on *Pulicaria* *undulata* which revealed promising antiangiogenic agents. Six compounds were isolated and identified as xanthoxyline (**1**), stigmasterol (**2**), oleanolic acid (**3**), salvigenin (**4**)**,** rhamnetin (**5**) and dihydroquercetin-4′-methyl ether (**6**) using nuclear magnetic resonance (NMR) spectroscopic techniques. Compound **3** and **4** are first reported in *Pulicaria* genus. Both the extract and isolated compounds were evaluated for in vitro antiproliferative activity against breast cancer cell line (MCF-7). In vivo antiproliferative activity against Ehrlich’s ascites carcinoma (EAC) were also assessed. The *P. undulata* extract and isolates showed significant reduction in tumor weight, decreased both serum vascular endothelial growth factor B (VEGF-B) levels and vascular endothelial growth factor receptor 2 (VEGFR-2) expression significantly compared to the control EAC group, suggesting an antiangiogenic activity through the inhibition of VEGF signaling. Besides, they displayed reduction in CD34 expression, confirming their antiangiogenic effect. Moreover, the potential affinity of isolated compounds to human estrogen nuclear receptor-alpha (*h*ER-α), the most recognized modulator of VEGFR-2 expression, was virtually estimated through molecular modeling studies. The most promising activity profiles were assigned to the investigated flavonoids, compounds **4**–**6**, as well as the alkyl-phenylketone, compound **1**. Additionally, these four top active compounds showed respective high to intermediate docking scores while possessing preferential binding with *h*ER-α critical pocket residues. Based on the provided data, these isolated compounds illustrated promising inhibitors of VEGF-stimulated angiogenesis, which could be a possible mechanism for their anticancer activity.

## 1. Introduction

Natural products have afforded a valuable source of diverse compounds that have found many applications in the fields of medicine and pharmacy [1]. Within the sphere of cancer, novel bioactive secondary metabolites of different chemical classes, and with different mechanisms of action, have been found as potential lead antitumor molecules isolated from plants, marine flora and microorganisms [2]. Cancer is among the main causes of morbidity and mortality over the world and is characterized by uncontrolled cell proliferation leading to the growth of abnormal cells that metastasize and invade to other parts of the body [3]. Thus, there is no doubt that there is a paramount need for new, efficient, improved cytotoxic agents with lower side effects, and plants are a promising source for such entities [1]. Cancer chemotherapy is disposed towards using more convenient drugs and a selective that can kill malignant tumor cells without affecting normal cells; many anticancer agents or their analogues currently in use are naturally derived and many more are under clinical trials [2]. Breast cancer is the most frequent invasive cancer in women and the second leading cause of cancer death among women after lung cancer; it is curable in ~70–80% of patients at an early stage [4]. The renewed interest in the discovery of natural anticancer drugs, especially from plant sources, has gained global attention. Among these natural molecules, numerous in vitro and in vivo studies have shown that flavonoids exert immunomodulatory, anti-inflammatory [5] and anticancer activities [6,7,8,9,10]. However, the main molecular mechanisms of these untapped natural compounds that are responsible for their activity have not been well elucidated yet. Sudhakaran *et al.* have illustrated the structure–functional impact of flavonoids and their effect on cancer cells, and emphasized their promising clinical role in the treatment and prevention of breast cancer [11]. Therefore, more investigations are required, increasing our understanding of flavonoid structure and molecular activities. Genus *Pulicaria* (Family Asteracaeae) is represented by about 80 species with a distribution from Europe to North Africa and Asia, especially around the Mediterranean [12]. Significant biological activities have been reported for *Pulicaria* sp. such as antispasmodic [13], antioxidant and cytotoxic activity [14], antimicrobial activity [15] and antihistaminic activity [16]. Previous phytochemical investigation on *Pulicaria* sp. revealed the presence of bioactive chemical constituents of different classes within the genus [16], such as sesquiterpene lactones [17], flavonoids and phenolics [18], mono-, di- and tri-terpenes [13] and steroids [16]. Most importantly, a number of compounds from *Pulicaria* species have been found to possess potent bioactivities; they could be promising candidates for the development of potential drugs and value added products. Here, within the presented study, we aimed to identify promising anticancer agents from the natural resources. Hopefully, the isolated bioactive metabolites could serve as lead candidates guiding the drug discovery and development of novel anticancer therapeutics, as well as facilitating their clinical translation.

## 2. Materials and Methods

### 2.1. General Experimental Procedures

The ^1^H NMR and ^13^C NMR spectra were recorded using Bruker high performance AVANCE III™ HD NMR spectrometer 300 MHz using CD_3_OD, CDCl_3_ and DMSO-*d_6_* as solvent. Column chromatographic separations were performed on Silica gel (60–120) mesh (Fluka) for fractionation obtained from Sigma-Aldrich chemicals, Darmstadt, Germany, flash silica gel (230–400) mesh was obtained from Whatman, Darmstadt, Germany, and Silica gel (70–230) was obtained from (Merk, Darmstadt, Germany) for column chromatography. Sephadex LH-20, for final purification, was purchased from Pharmacia Fine Chemicals AB (Uppsala, Sweden), Merck Pre-coated Silica 60 GF245 plates (20 cm × 20 cm) (Darmstadt, Germany) were used for thin layer chromatography TLC. Compounds were detected by UV absorption at λmax 255 and 366 nm, followed by spraying with p-anisaldehyde/H_2_SO_4_ reagent and heating for 2 min at 110 °C.

### 2.2. Collection of Plant Materials

*Pulicaria undulata*, plant growing wild in Egypt in the desert of North Sinai [19], was collected in March 2018 from Sinai Peninsula in Egypt. The whole plant was air dried in the dark and then crushed into fine powder by mechanical grinders and stored at low temperature until extracted with methanol. The plant was kindly authenticated as Pulicaria undulata (Asteraceae) by Prof. Dr. El-Sayeda Gamal El-Din, Faculty of Science, Suez Canal University, Egypt. The voucher specimen was deposited in the herbarium section of Pharmacognosy Department, Faculty of Pharmacy, Suez Canal University, Ismailia, Egypt under registration number SAA-143.

### 2.3. Extraction and Purifications of Compounds ***1**–**6***

The plant powder was extracted with 95% methanol and concentrated to give a residue (150 g). Fractionation was carried out by subjecting the concentrated alcoholic extract to successive fractionation using vacuum liquid chromatography (VLC). The column was eluted with different organic solvents (n-hexane, EtOAc and MeOH) giving five successive fractions, then each dried by evaporating solvent using rotary evaporator to give, finally, 2 g of F1 (100% n-hexane), 17 g of F2 (50% n-hexane in EtOAc), 15 g of F3 (100% EtOAc), 20 g of F4 (50% EtOAc in MeOH) and 30 g of F5 (100% MeOH).

Fraction F2 and F4 were selected based on bioassay guided fractionation of the active extract of *Pulicaria undulata* with IC_50_ values at 32.30 and 12.70 µg/mL, respectively. F2 and F4 under study were purified on silica gel and sephadex LH-20 columns as following: F2 (17 g) adsorbed on silica gel, rechromatographed over silica gel column (250 g × 120 cm × 3 cm ∅) using *n*-hexane 100% then EtOAc/n-hexane (2:98) added; elution afforded subfractions showed two major spots which were investigated by TLC and were further purified by rechromatography on flash silica gel column (50 g × 80 cm × 2 cm ∅) using EtOAc/*n*-hexane (3:97) to give compound **1** (60 mg) and compound **2** (90 mg). Fraction **4** (20 g) revealed four major compounds by TLC investigations which were purified by rechromatography on silica gel column (350 g × 150 cm × 3 cm ∅) using EtOAc/*n*-hexane (10:90) to 100% EtOAc; elution afforded 3 subfractions (F4-A, F4-B, F4-C); then F4-A (3 g) was washed by MeOH white powder and was recrystallized to give compound **3** (110 mg). Repeated purification of F4-B, F4-C using flash silica gel column and Sephadex LH-20 (20 g × 80 cm × 2 cm∅) using Methanol: dichloromethane (1:1) as isocratic eluent afforded 3 major compounds: **4** (55 mg), compound **5** (70 mg) and, finally, compound **6** (65 mg). Then, NMR experiment was performed to elucidate the structures of the compounds which were then compared with literature as H^1^ and C^13^ signals were in accordance with that of the references mentioned in the supplementary data.

### 2.4. In Vitro Assessment of the Antiproliferative Activity of the Total Extract and Isolated Compounds ***1**–**6***

Sulphorhodamine B (SRB) colorimetric assay, the most widely used method for in vitro screening of the antiproliferative activity, has been used for the evaluation of potential antiproliferative activities of the extract and isolated compounds on breast cancer cell line (MCF-7), as described before [20]. In brief, cell lines were seeded in a 150 µL fresh medium for 24 h in 96-well microtiter plates. Then, the test samples were dissolved in dimethyl sulfoxide and added at concentrations of 0, 5, 12.5, 25, 50 µg/mL. After incubation for 48 h, the cells were fixed with 50 μL of 10% trichloroacetic acid and incubated for 60 min at 4 °C. The plates were washed and stained with 50 μL of 0.4% SRB dissolved in 1% acetic acid for 30 min at room temperature. Then, the plates were air-dried and the protein bound dye was solubilized with 100 μL/well of 10M tris base (PH 10.5) (Applichem, Darmstadt, Germany). Optical density (O.D.) of each well was determined spectrophotometrically at 570 nm with ELISA microplate reader (Sunrise Tecan reader, Deutschland GmbH, Germany). The absorbance was subtracted and mean values of each sample concentration were calculated. The experiment was repeated 3 times and IC_50_ values were calculated using doxorubicin as positive control. The tumor cell line (MCF-7) was obtained from the National Cancer Institute, Cairo, Egypt. The cells were subcultured on RPMI 1640 medium supplemented with 1% penicillin/streptomycin and 10% fetal bovine serum (Sigma Aldrich Chemical, Darmstadt, Germany).

### 2.5. In Vivo Assessment of the Antiproliferative Activity of the Total Extract and Isolated Compounds ***1**–**6***

#### 2.5.1. Induction of EAC Solid Tumors

Cells of EAC were purchased from Tumor Biology Department, National Cancer Institute, Cairo University. EAC is a murine spontaneous breast cancer that served as the original tumor from which an ascites variant was obtained. Ascetic fluid was aspired under aseptic conditions from the peritoneal cavity of tumor-bearing mice. The viability and contamination of EAC cells were tested using trypan blue dye exclusion technique. Trypan blue dye stains dead cells only. EAC cells with 90% viability or more were suspended in normal saline (2.5 × 10^6^ cells/0.1 mL). The mice were inoculated intradermally with 0.l ml EAC suspension on the lower ventral side after shaving.

#### 2.5.2. Study Design

Swiss albino female mice obtained from the Egyptian Organization for Biological Products and Vaccines (Vacsera, Egypt), and weighing 25–30 g were used. The mice were kept in plastic cages with mesh floor and hardwood bedding at 25 °C temperature under normal light/dark cycle, with water and food provided ad libitum. Mice were left to acclimatize for 1 week before the experiments. They were randomly divided into nine groups (7 mice each). The first group served as the normal group and received saline. EAC cells were inoculated in all other groups. Group 2 was considered the EAC control. Group 3 received (200 mg/kg) of *P. undulata* extract PO three times weekly. Groups (4–9) received (50 mg/kg) of Xanthoxyline, Oleanolic acid, Stigmasterol, Salvigenin, Rhamnetin and Dihydroquercetin 4′-methyl ether, respectively, PO three times daily. All compounds were dissolved in saline.

The day of tumor inoculation was considered as day zero (0). Treatment of groups (3–9) started at the 7th day after tumor inoculation, and continued for 2 weeks until day 21. The in vivo study was in accordance with the Guide for the Care and Use of Laboratory animals. The study protocol was approved by the ethical committee of Faculty of Pharmacy, Suez Canal University (code of approval 20201907RA2)

#### 2.5.3. Sample Collection

On the 21st day of EAC cells inoculation, blood samples were collected under light ether anesthesia from the orbital sinus (retro-orbital plexus). The blood samples were left to clot for 20 min followed by separation of the serum by centrifugation at 4000 rpm for 15 min. Mice were sacrificed by cervical dislocation and tumor discs were separated, weighed and fixed in 10% neutral buffered formalin for immunohistochemical investigations.

#### 2.5.4. Determination of Vascular Endothelial Growth Factor B (VEGF-B)

Serum samples were stored at (−20 °C) and used for determination of the level of VEGF-B by ELISA kit (ab213897) (Abcam, Cambridge, UK) according to the manufacturer’s protocol.

#### 2.5.5. Immunohistochemistry

For immunohistochemical examination, the tumor discs were fixed in 10% neutral buffered formalin overnight and then embedded in paraffin. When analyzed, all paraffin-embedded tissues were sectioned at 4-μm, deparaffinized in xylene, and hydrated through graded ethyl alcohol series in decreasing concentrations (100%, 90%, 80% and 70%). Antigen retrieval was performed according to Tris/EDTA buffer (pH = 9) antigen retrieval protocol. Tumor discs were stained applying the EnVision™ FLEX HRP labeled, High pH, method according to the manufacturer’s staining protocol (Dako Omnis, Santa Clara, CA, USA). Primary polyclonal antibodies for VEGFR-2 and CD34 (Santa Cruz, CA, USA) were diluted in PBS at a ratio of 1:250. Finally, Mayer’s hematoxylin was used for counter staining. ImageJ was used for the semiquantitative analysis of immunohistochemical reactions. The images were captured by an optical microscope with a 40X objective (Optika B-352A, Ponteranica (BG), Italy) coupled to a camera (HDCE30C). The expression of VEGFR-2 and CD34 were assessed and percentage of stained area was measured.

### 2.6. Molecular Docking Studies

The docking studies of the prepared ligands into binding cavity of hER-α LBD (PDB ID: 1ere; atomic resolution 3.10 **Å**) was performed using molecular operating environment (MOE) software [21]. Preparation of both ligands and target were proceeded according to reported routine protocol [22,23]. In brief, investigated ligands were constructed and energy minimized utilizing the MMFF partial charges and MMFF (modified) force field with 2000 steps of conjugate gradient method until 1 × 10^−3^ Kcal/**Å** gradient. The biological target prepared through 3D protonation, termini capping, and auto-correction for atoms types, connections, and charges. The binding site was defined by MOE Alpha Site Finder then the active site was refined so as to involve the crucial residues being reported within literature. Dummy atoms were generated from the obtained alpha spheres as hydrophobic-polar descriptors for the defined hER-α binding site. Examining the B-factors of binding site revealed significant rigidity of its constituting residues, which rationalized the development of rigid docking protocol. Initially, the validity of the adopted docking protocol was confirmed through redocking the cocrystallized ligand (E2; PDB ID: est) within the target protein binding site. Interestingly, the validation stage illustrated a root-mean-square deviation (RMSD) of 0.9920 **Å** for the redocked cocrystallized ligand. Clearly, depicting RMSD values below 2 **Å** indicates that both the adopted algorithms and parameters were sufficient for determining the best docking pose [24]. Following validation, the docking protocol was proceeded for the investigated compounds. Throughout the molecular docking study, ligand conformations were developed through bond rotation, lodged within in defined binding site guided by the triangle matcher method, and then ranked using London dG scoring function. Sets for 10 poses per ligand were then passed to the refinement process before rescoring with generalized born solvation model/Weighted Surface Area (GBVI/WSA) dG forcefield-based scoring module. Achieving significant interactions with key pocket residues as well as furnishing RMSD values below the threshold 2.0 **Å** were both considered for assessing docking pose validity and selection. PyMol v2.0.6 was used for the analysis and final visualization of ligand–target interactions.

### 2.7. Statistical Analysis

Data were presented as mean ± standard error of mean (S.E.M). One-way ANOVA followed by Bonferroni’s posthoc test were used to analyze the difference between the experimental groups. All statistical tests were performed using SPSS 22. A *p*-value < 0.05 was considered to be statistically significant.

## 3. Results and Discussion

### 3.1. Isolation and Identification of P. undulata Metabolites ***1**–**6***

**Figure 1:**
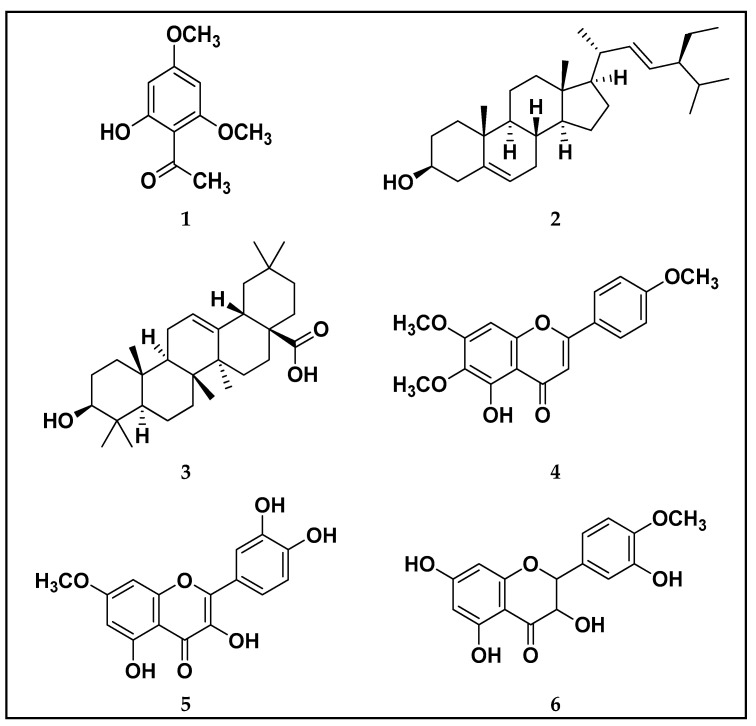
Structures of the isolated compounds **1**–**6**.

Successive chromatographic fractionation of the combined extracts of *P.* *undulata* resulted in the isolation and purification of compounds **1**–**6** (Figure 1). The identification and structure elucidation of the isolated compounds was deduced on the basis of the NMR spectroscopic method (Supplementary Materials, Figures S1–S18), physicochemical properties, in addition to comparison with literature data and/or authentic samples. The compounds were identified as Xanthoxyline (2-hydroxy-4, 6-dimethoxyacetophenone) (**1**) [16], Stigmasterol (**2**) [25], Oleanolic acid (**3**) [26], salvigenin (5-hydroxy-4’,6,7-trimethoxyflavone) (**4**) [27], rhamnetin (5,3’,4’-trihydroxy-7 methoxyflavonol) (**5**) [28], dihydroquercetin 4′-methyl ether (**6**) [29]. To the best of our knowledge compounds **3** and **4** are the first isolated from the *Pulicaria* genus

### 3.2. In Vitro Antiproliferative Activity of the Total Extract and Isolated Compounds ***1**–**6***

The potential cytotoxic activities of the *P. undulata* extract and isolated compounds against breast cancer cell line (MCF-7) were evaluated using a Sulphorhodamine-B (SRB) assay. The IC_50_ values of the total extract and isolated compounds (the concentration required for 50% of growth inhibition) on the MCF-7 cell line and normal cell lines were calculated, as shown in Table 1.

The crude extract of *Pulicaria undulata* displayed significant cytotoxic activity on the MCF-7 cell line with inhibition of 68% (IC_50_ 28.10 ± 0.17); Methoxyflavonoids, compounds **4**–**6**, as well as the alkyl-phenylketone Xanthoxyline, compound **1**, have displayed the most remarkable cytotoxic activity on the MCF-7 cell line with IC_50_ values at 18.00, 18.50, 22.50, and 23.50 µg/mL, respectively. Our results showed that the isolates showed promising anticancer activity, moreover, the extracts, along with compounds isolated, showed a weak antiproliferative activity on cells of the normal Vero cell line.

### 3.3. In Vivo Antiproliferative Evaluation of the Total Extract and Isolated Compounds ***1**–**6***

#### 3.3.1. Tumor Weight

**Figure 2:**
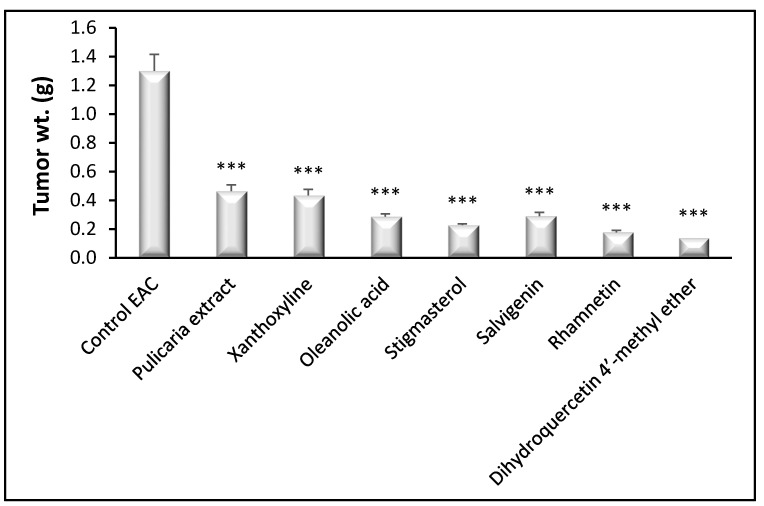
Effect of *P. undulata* extract and the six isolated compounds on weight of EAC solid tumors growing in female mice. Data are expressed as the mean ± S.E.M and analyzed using one way ANOVA followed by Bonferroni’s posthoc test. Significance was set at *** *p* < 0.001 as compared to the control EAC group.

Solid tumor discs of Ehrlich ascites carcinoma (EAC) were separated and weighed for evaluation of the antitumor activity of the *P. undulata* extract and the isolated compounds. Treatment with the *P. undulata* extract and all other compounds presented a significant reduction in tumor weight (*p* < 0.001) compared to the control EAC. Interestingly, the least tumor weights were observed with rhamnetin (**5**) and dihydroquercetin-4′-methyl ether (**6**) treated groups (Figure 2).

#### 3.3.2. Effect on VEGF-B and VEGFR2

**Figure 3:**
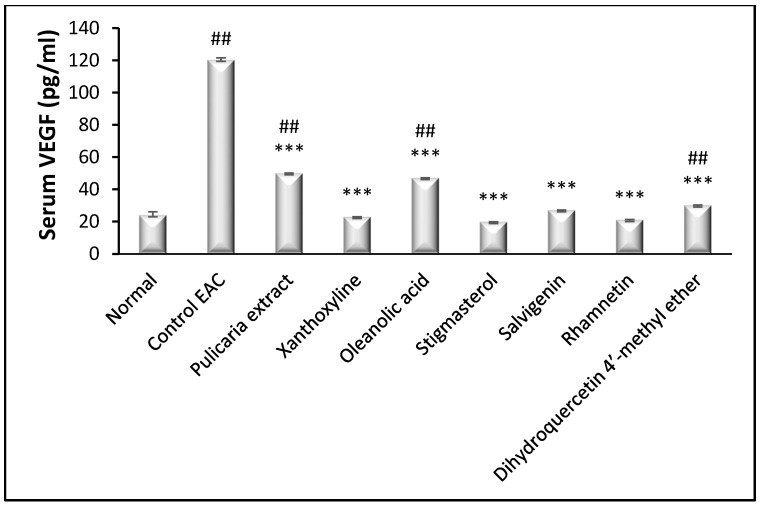
Effect of *P. undulata* extract and the six isolated compounds on serum VEGF levels in female mice with EAC. Data are expressed as the mean ± S.E.M and analyzed using one way ANOVA followed by Bonferroni’s posthoc test. Significance was set at *** *p* < 0.001 as compared to the control EAC group and at ## *p* < 0.01 as compared to normal group.

Assessment of the serum levels of VEGF-B revealed a marked increase in control EAC group compared to normal. Treatment with the *P. undulata* extract and all other compounds decreased serum VEGF-B levels significantly (*p* < 0.001) compared to the control EAC group. It is worth mentioning that xanthoxyline (**1**), stigmasterol (**2**), salvigenin (**4**) and rhamnetin (**5**) treated groups could nearly normalize VEGF-B levels (Figure 3).

**Figure 4:**
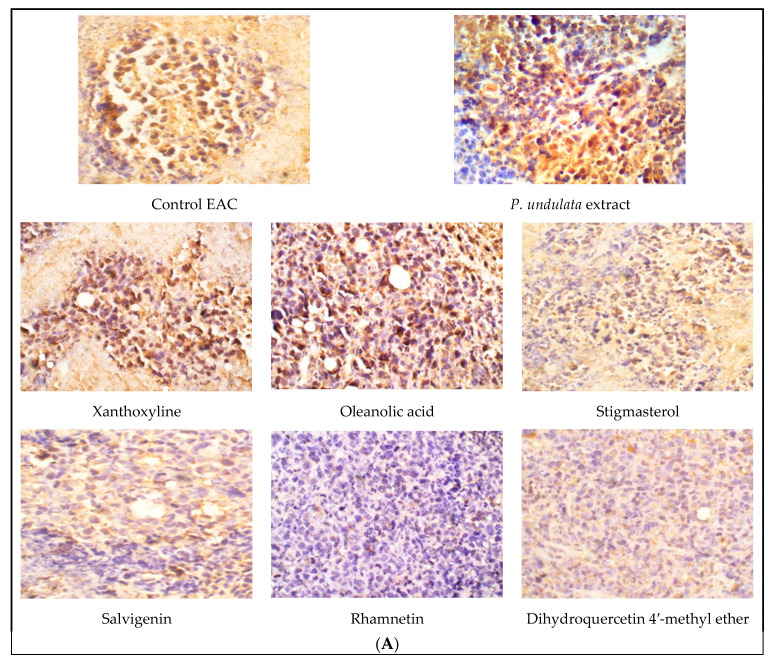

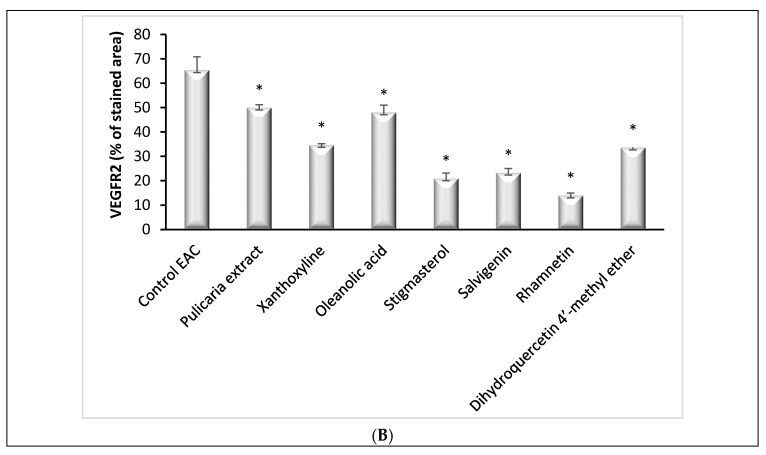
Effect of *P. undulata* extract and the six isolated compounds on VEGFR-2 expression in tumor tissue. (**A**) Representative photomicrographs of VEGFR-2 expression; (**B**) the percentage of positive immunohistochemical reactions (brown stained area) analyzed by Image J software. Data are expressed as the mean ± S.E.M and analyzed using one way ANOVA followed by Bonferroni’s posthoc test. Significance was set at * *p* < 0.05 as compared to the control EAC group.

Immunohistochemical analysis of VEGFR-2 displayed high expression in the control EAC group. The *P. undulata* and oleanolic acid (**3**) groups significantly (*p* < 0.05) lowered VEGFR-2 expression compared to the control EAC group. All other compounds showed diminished expression with significant (*p* < 0.001) differences from the control EAC. The lowest expression was observed with the rhamnetin group (Figure 4A,B).

Breast cancer cells need constant nourishment and an oxygen supply through the vascular network of capillaries. Consequently, angiogenesis, the fast increase in blood vessels formation, is essential for the supply of sufficient oxygen and nutrition for breast tumor growth [30]. The initiation and progression of tumor angiogenesis are mainly due to angiogenic growth factors, such as VEGF and fibroblast growth factors (FGF) [31]. It has been reported that levels of angiogenic factors, and the subsequent number of vascular networks formed, is a predictive factor for breast cancer survival [32]. Therefore, compounds that target the angiogenesis pathway have progressively attracted attention in research in breast cancer treatment [33].

The plasma level of VEGF-B is a sensitive marker in breast cancer [34]. VEGF signaling involves the binding of the VEGF ligand to receptor tyrosine kinases VEGFR-1, VEGFR-2 and VEGFR-3, resulting in angiogenesis, vasculogenesis and lymphangiogenesis [35]. VEGF mainly activates a VEGFR-2-mediated signaling cascade to induce angiogenic responses. These signaling pathways include the activation of the phospholipase C-γ (PLC γ)– Extracellular signal regulated protein kinase (ERK1/2) pathway; phosphatidylinositol 3-kinase (PI3K) and its downstream molecule serine/threonine protein kinase B, known as AKT-mammalian target of rapamycin (PI3K–AKT–mTOR) pathway; the Proto-oncogene tyrosine protein SRC kinases and guanosine triphosphate hydrolases (small GTPases) pathways that are involved in cell survival and migration, besides the regulation of endothelial junction and vasomotion during vascular development [36]. Moreover, VEGF signaling through R2 promotes endothelial nitric oxide synthase (eNOS). This eNOS produces nitric oxide (NO), an important vasodilator that rapidly diffuses throughout the endothelium and activates the enzyme soluble guanylate cyclase (sGC) to produce cyclic guanosine monophosphate (cGMP). This particular part of the eNOS signaling pathway contributes to both an acute vasodilating effect in the neighboring vascular smooth muscle cells (VSMCs) and the long term angiogenic functions of ECs, such as proliferation [37].

Assuming the crucial role of VEGFR-2 signaling in angiogenesis, regulation of VEGFR-2 activation may represent an important mechanism for the control of angiogenesis [38]. Hence, inhibition of VEGF signaling results in angiogenesis inhibition and decrease in tumor growth. In the present study, the inhibition of VEGF-B and VEGFR-2 mediated by the *P. undulata* extract and the isolated compounds suggests an antiangiogenic activity through the deactivation of VEGF signaling. This may provide evidence for the mechanism of the antiproliferative activity of the extract and isolated compounds. Regarding immunohistochemical analysis of VEGFR-2 expression, treatment with the crude extract showed a significant decrease in VEGFR-2 expression compared to the control EAC group, indicating the antiangiogenic activity of the extract through the inhibition of the VEGF/VEGFR-2 system. This result coincides with the findings of in vitro studies which proved the cytotoxic activity of the extract and may provide an explanation for the mechanism of this cytotoxic activity.

#### 3.3.3. Effect on CD34

**Figure 5:**
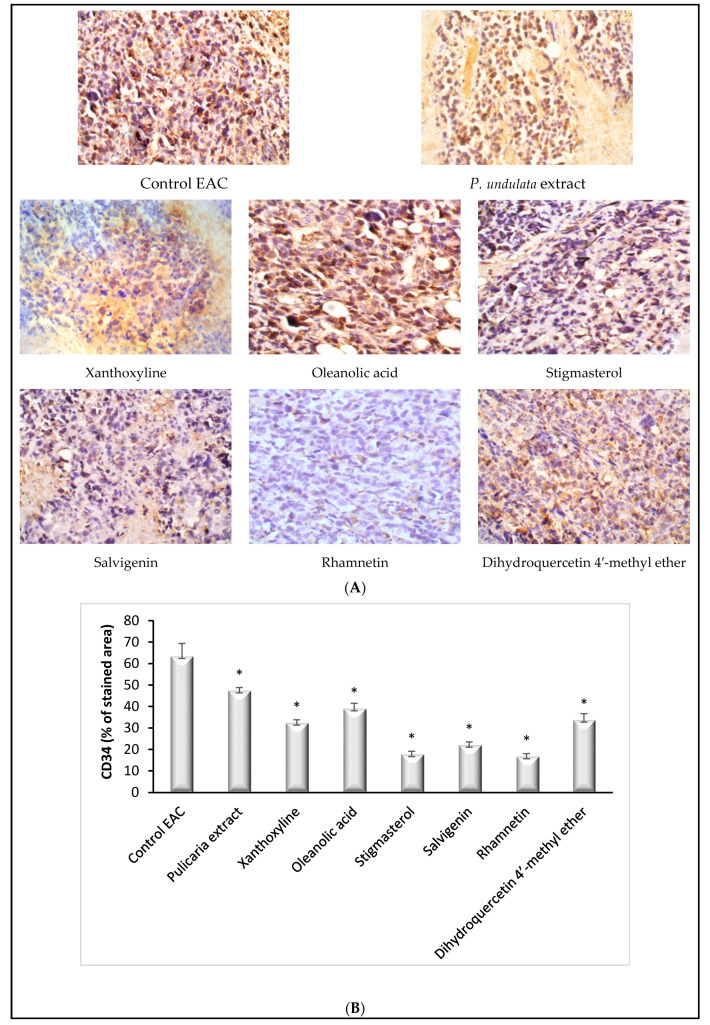
Effect of *P. undulata* extract and the six isolated compounds on CD34 expression in tumor tissue. (**A**) Representative photomicrographs of CD34 expression; (**B**) the percentage of positive immunohistochemical reactions (brown stained area) analyzed by Image J software. Data are expressed as the mean ± S.E.M and analyzed using one way ANOVA followed by Bonferroni’s posthoc test. Significance was set at * *p* < 0.05 as compared to the control EAC group.

The immunohistochemical analysis of CD34 demonstrated high expression in the control EAC group. Treatment with the *P. undulata* extract significantly (*p* < 0.05) reduced CD34 expression compared to the control EAC group. Treatment with all the isolated compounds decreased the expression, with significant (*p* < 0.001) difference from the control EAC group (Figure 5A,B). Treatment with rhamnetin showed the least expression of CD34. Furthermore, groups treated with stigmasterol and salvigenin showed poor expression of CD34.

Angiogenic activity can be detected by measuring the expression of diverse molecules involved in angiogenesis, such as CD31 and CD34 [39]. CD34 is a transmembrane antiadhesive sialomucin that is expressed in the luminal surface of endothelial cells [40]. The CD34 antigen has been widely used in studying tumor angiogenesis [41]. A role of CD34 in tumor angiogenesis was proposed by the observation that CD34 deletion in mice impaired early tumor growth due to a delay in angiogenesis [42]. A high expression of CD34 on endothelial tip cells and their filopodia suggests its important role in angiogenesis. In the current study, reduction of CD34 expression by the *P. undulata* extract and the isolated compounds confirms their antiangiogenic activity.

Based on the above biological results, the isolated compounds showed a significant impact on the angiogenesis pathway within cancer cells at different levels. Affecting key modulators such as VEGFR2 and VEGF-B, in addition to influencing the expression of transmembrane antiadhesive sialomucin (CD-34), the isolated compounds highlighted the synergism conceptualization as the multi-target activity of natural metabolites [43]. Moreover, the obtained antiangiogenesis activity of these isolated bioactive compounds have confirmed their respective pleiotropic effect being anticancer agents, rather than only dietary nutrients or acting as free radical scavengers [44,45]. It has been suggested that the molecular promiscuity and character diversity acquired through evolution by the naturally occurring bioactive metabolites have allowed them to modulate multiple molecular pathways and targets as well [46]. It is worth mentioning that the obtained anticancer biological activity of the examined *Pulicaria undulata* extract could be further correlated to the nonisolated compounds rather than just the major ones. The latter concept could be reasoned since such minority isolates could further participate within the synergistic effect that build up the total activity of the plant.

### 3.4. Computational Investigation

Consolidating the differential anticancer activity of the investigated compounds on the MCF-7 cell line was proceeded through a highly validated computational investigation on the human estrogen receptor-alpha (*h*ER-α). Currently, the transmembrane receptor, VEGFR-2, is highly recognized for playing a key role within endothelial cell development, proliferation, and permeability, exhibiting actions significantly related to VEGF-stimulated angiogenesis [47,48,49]. Previous reports also showed that VEGFR-2 was expressed in mammary tumors and both *h*ER-negative and -positive breast cancer cell lines, including T47D and MCF-7 cells [50,51,52,53,54,55,56,57,58,59]. Regulation of VEGFR-2 expression is complex and proceeded through different pathways, with the presence of estrogens as one of the most important modulators. Treating MCF-7 breast cancer cells with estrogen showed the down regulated expression of several genes, including that of VEGFR-2 [60]. Higgins et al. have explored the molecular mechanistic aspects behind the estrogen depended, down regulation of VEGFR-2 within MCF-7 cells [61]. MCF-7 is the breast cancer cell model expressing *h*ER-α, rather than the other isoform *h*ER-β [62]. The study by Higgins and research group illustrated that the binding of *h*ER-α, in complex with transcription factor (Sp), at the critical GC-rich sites at −58 and −44 of the VEGFR-2 promotor, was crucial for the down regulation responses. The *h*ER-α/Sp complex was formed by the Af-1, Af-2 and DNA binding domain of *h*ER-α being linked to Sp, where the latter is being directly attached to the GC-rich site. In the absence of a ligand, the *h*ER-α/Sp complex is constitutively associated with a VEGFR-2 promotor, mediating the protein and mRNA basal expressions within MCF-7 cells. However, upon estrogen treatments, the nuclear corepressors, NCoR (nuclear receptor corepressor) and SMRT (silencing mediator of retinoid and thyroid hormone receptor), are recruited for depressing the transactivation processes. Based on the above evidence, the capability of the isolated compounds to accommodate the estrogen binding site at *h*ER-α while exhibiting a preferential binding mode comparable to the endogenous substrate can correlate to the depicted antiproliferative activity on MCF-7 cells.

Interestingly, several isolated flavonoids have shown selective modulation of *h*ER-α signaling pathways [63,64,65,66]. Long et al. investigated the anticancer activity of the apigenin flavonoid for the growth inhibition of antiestrogen resistant breast cancers [67]. Interestingly, high apigenin concentrations exhibited *h*ER-α-independent and -dependent mechanisms through suppressing *h*ER-α mobilization, downregulating expressions of *h*ER-α/amplified in the breast cancer-1 co-activator, and blocking several protein kinases, such as protein kinase A (PKA), p38, AKT, and MAPK. Similarly, naringenin and quercetin antagonized *h*ER-α rapid signaling, yet with persistent activation of p38, AKT inhibition, extracellularly regulated kinase-1/2 E2-dependent activation and no *h*ER-α transcriptional mechanism [68,69]. Several other polyphenolics showed relevant binding to *h*ER-α, exhibiting biological activity relative to cancer cell proliferation inhibition [63,70,71,72]. Notably, plant sterols, stigmasterol, have shown possible interfere with E2 action within cultured human breast and endometrium cell lines [73]. The latter evidence further consolidates the computational investigation of the potential binding affinities of *P. undulata* isolated compounds towards *h*ER-α receptors for evaluating the differential binding of presented drugs with *h*ER-α in a way to explain the furnished cytotoxic biological activity.

**Figure 6:**
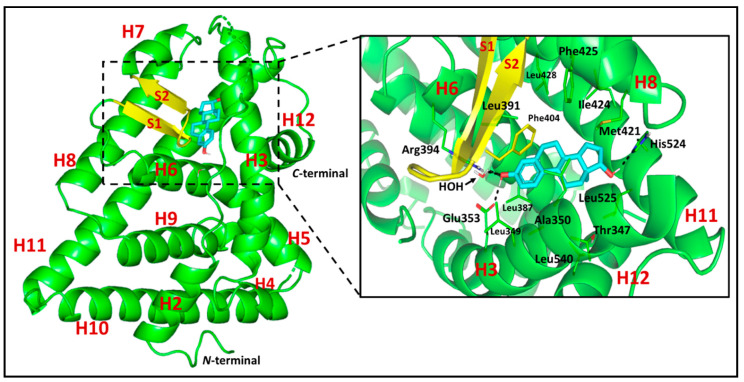
Cartoon 3D-representation of X-ray crystallized *h*ER-*α* LBD (PDB ID: 1rer) bounding to steroidal endogenous ligand, 17*β*-estradiol (E2; cyan sticks). The *h*ER-*α* LBD indicates the location of its secondary structures, H1→12 *α*-helices (green), loops (green), and extended region of anti-parallel β-sheet (S1/S2; yellow), which are numbered according to former established nomenclature of other nuclear receptor LBDs. Zoomed image is a stereoview of crystallized E2 (cyan sticks) occupying the canonical binding site of *h*ER-*α* across H3, H6, H8, H11, and H12. Polar interactions (Hydrogen bonding) are illustrated as black dashed lines. Only residues (lines) located within 4 **Å** radius of bound ligand are displayed and labeled with sequence number.

In this presented molecular docking study, the 3.10 **Å** X-ray crystallographic structure of *h*ER-α, bounded to its steroidal endogenous ligand 17β-estradiol, was acquired from Research Collaboratory for Structural Bioinformatics (PDB ID: 1ere) (Figure 6) [21]. The target’s binding cavity occupies a large hydrophobic portion of its ligand binding domain (LBD), being highly obscured from the external environment. The pocket lies within parts of H3, H6, H8, H11, H12, and S1/S2 hair pin, where E2 diagonally occupies the binding cavity between H3, H6, and H11, adopting favored low energy conformation. Ligand recognition is mediated through a combination of definite polar interactions and hydrophobic contacts, being highly complementarity with the ligand’s nonpolar nature. Hydrogen bonding between the ring-A phenolic-OH and each of Glu353, Arg394, and a crystallized water molecule is depicted at H3 and H11 of the pocket. Direct polar interaction with His524 of H11 is recognized for the ring-D 17β-OH, acting as hydrogen-bond donor. The extended hydrophobic skeleton of E2 is involved within several nonpolar contacts with pocket lining residues, Ala350, Leu387, Leu394, Phe404, Met421, Ile424, Phe425, Gly521, Leu525, and as being condensed around the A, A/B interface, and D-rings. Interestingly, Phe404 is at a perpendicular plane relative to E2’s ring-A. The presence of extra unoccupied subpockets, against the α- and β-faces of ring-B and -C, respectively, can account for the binding site’s ability to accommodate various numbers of branched scaffolds.

Initially, the validity of the adopted docking protocol was confirmed through redocking the cocrystallized ligand (E2; PDB ID: est) within the target protein binding site. Interestingly, the validation stage illustrated a root-mean-square deviation (RMSD) of 0.9920 **Å** for the redocked cocrystallized ligand, indicating that both the adopted algorithms and parameters were sufficient for determining the best docking pose (Table 2). Throughout the adopted docking protocol, the flavonoids, salvigenin (**4**), rhamnetin (**5**), and dihydroquercetin-4′-methyl ether (**6**), showed the highest docking ranks (*S* = −11.067 to −15.479 Kcal·mol^−1^), followed by the alkyl-phenylketone, with xanthoxyline an intermediate docking score (−6.301 Kcal·mol^−1^) (Table 2). The latter findings is in good agrement with the above biological studies, where the most promising MCF-7 growth inhibition activity profiles were assigned to compounds **1** and **4**–**6**, as compared to the investigated plant sterol; stigmasterol and triterpene; oleanolic acid.

**Figure 7:**
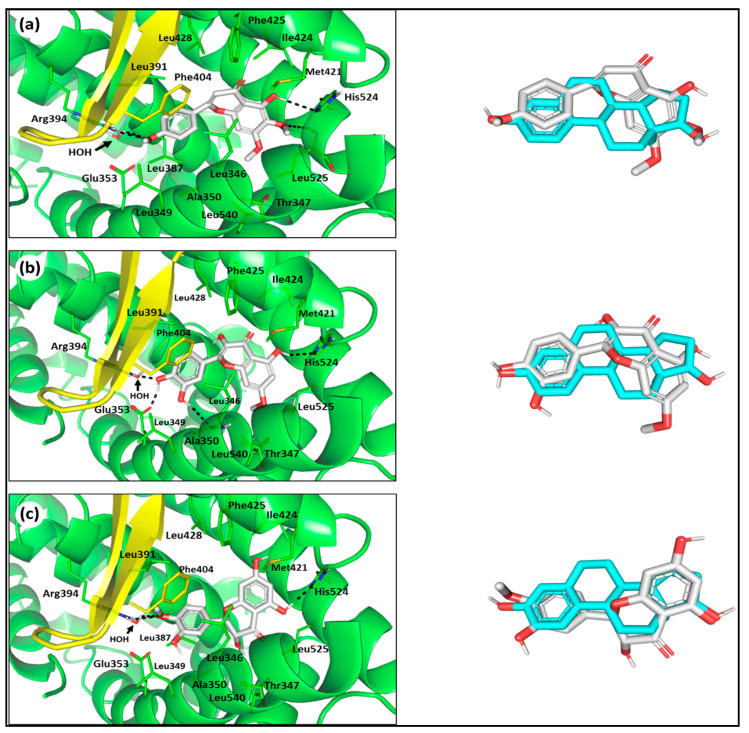
Molecular docking investigation of the investigated compounds: (**a**) salvigenin; (**b**) rhamnetin; (**c**) dihydroquercetin-4′-methyl ether, isolated from *P. undulata* at *h*ER-*α* LBD binding site (PDB ID: 1ere). The left panels illustrate predicted binding modes of the target compounds (white sticks) at green cartoon 3D representation of the biological target. On the right, overlay of the docked compounds and crystallized ligand (E2; cyan sticks), depicting their comparative orientations within the binding site. Polar interactions (Hydrogen bonding) are illustrated as black dashed lines. Only residues (lines) located within 4 **Å** radius of bound ligand are displayed and labeled with sequence number.

Examining the predicted docking poses of the top ranked flavonoids predicted a comparable extended orientation within the *h*ER-α binding site. The latter orientation permitted significant polar interactions with key conserved residues at the opposite sides of the occupied pocket. The three promising flavonoids exhibited the “E2’s pincer-like arrangement” around their phenolic scaffold, allowing relevant insertion between Ala350 and Leu387 from one side and Phe404 at the other, favoring π-hydrogen interaction with Phe404 (Figure 7). This significant ligand orientation is characteristic for E2 ring-A and its effective analogues, permitting close ligand proximity towards Glu353, Arg394, and/or crystallized water for extended hydrogen bond networking. The phenolic scaffolds of top docked flavonoids, particularly for dihydroquercetin-4′-methyl ether (**6**), showed relevant superimposition with ring-A of the crystallized ligand. At the other end of the *h*ER-α binding cavity (H8, H11, and H12 site), the terminal oxygen functionality of the 4H-chromen-4-one core skeleton predicted significant hydrogen bonding with His524 and even the vicinity residue, Leu525. Further stabilization of flavonoid binding modes was predicted through hydrophobic contacts with pocket lining nonpolar residues (Leu346, Ile424, Phe425, Leu428, Leu525, and Leu540) along the 4H-chromen-4-one core skeleton. Despite the depicted comparable binding modes and docking scores of the three flavonoids, key structural–activity insights were deduced relating differential orientation/conformation and hydrogen bond networking for each flavonoid with the pocket’s key and vicinal residues.

The highest docking score was rhamnetin (**5**), with predicted extended polar interactions with the key residues, Glu353 and Arg394, at H3/H6 site. Moreover, the stabilization of rhamnetin (**5**) was further suggested through extra hydrogen bonding with the Leu346 mainchain. The latter preferential ligand–protein interactions were correlated for the rhamnetin incorporating double oxygen functionalities (OH and MeO as Hydrogen bond donor/acceptor) on its 2-phenyl moiety. On the other hand, salvigenin (**4**) possesses only single MeO at the para-position of its benzene ring and so predicted different and fewer polar networkings with the key pocket residues, lacking interactions with Glu353. Since MeO acts only as a hydrogen bond acceptor, salvigenin predicts polar interaction with the crystallized water rather than with the Glu353 key residue. Additionally, having this hydrophobic methyl group directed towards the key hydrophilic site can impose an additional hydrophobic penalty against the binding affinity of salvigenin, which correlated with the lowest docking score among the investigated flavonoids. Concerning dihydroquercetin-4′-methyl ether (**6**), this flavonoid also possesses double oxygen functionality (OH and MeO), where its MeO group predicted a similar pattern of interacting with crystallized water rather than with Glu353, as well as suggesting a hydrophobic penalty against the compound’s docking score. Nevertheless, the ligand’s meta-OH group showed a favoured direction towards the polar pocket residues, which can positively contribute into its docking score. Despite having meta-OH, the ligand failed to achieve polar interaction with the vicinal residue (Leu346) which can be due to the 2,3-single bond at the flavone scaffold. The lack of significant rigidity and planarity within the flavone ring could alter the orientation of the 2-phenyl group, allowing a relevant retraction of the meta-OH far from Leu346. All such findings contributed within the intermediate docking score of dihydroquercetin-4′-methyl ether as compared to the other investigated flavonoids.

Concerning the flavone ring interaction with the rest of the binding sites (H8, H11, and H12), the 4H-chromen-4-one core skeleton of all three flavonoids depicted significant hydrogen bond pair interaction between the flavone 4-OH and His524, while being also conserved with the crystallized ligand, E2. For dihydroquercetin-4′-methyl ether (**6**) to achieve the latter important polar interaction an inverted orientation of the flavone ring was depicted, as compared with those of other flavonoids, where the latter 1-oxo functionality was directed away from the S1/S2 pin. Such inverted orientation further contributed to the retracted position of the meta-OH group, hindering the polar interaction with Leu346. Thus, the unsaturation within the 4H-chromen-4-one core skeleton could negatively contribute to the flavonoid’s binding to the *h*ER-α binding site. It is worth noting that each investigated flavonoid actually possesses differential polar substitution patterns upon their respective flavone rings. These polar functionalities were of minimal contribution to both rhamnetin (**5**) and dihydroquercetin-4′-methyl ether (**6**) electrostatic binding, owing to their respective orientation towards the hydrophobic pocket lining residues (Leu346, Ile424, Phe425, Leu428, Leu525, and Leu540). This was with the exception of salvigenin (**4**), as its 6-MeO group afforded hydrogen bond interaction with the mainchain of the vicinal residue, Leu525, which can positively contribute to the ligand’s docking score. Moreover, possessing several MeO groups on the flavone ring, particularly with salvigenin (**4**), was considered beneficial for minimizing the electrostatic penalty and providing hydrophobic preferentiality towards the pocket lining nonpolar residues. Relying on the flavonoid structure related binding insights, the differential functionalities of the investigated flavonoids appear to positively and negatively contribute to the own ligand’s binding that arise in the somewhat comparable docking scores for the three investigated flavonoids.

**Figure 8:**
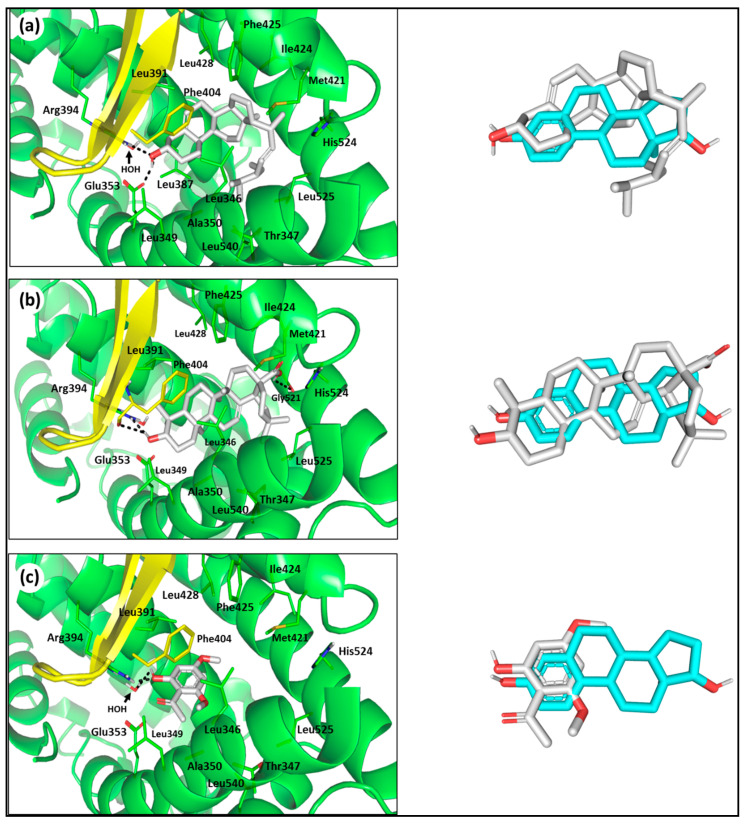
Molecular docking investigation of the investigated compounds: (**a**) stigmasterol; (**b**) oleanolic acid; (**c**) xanthoxyline, isolated from *P. undulata* at *h*ER-*α* LBD binding site (PDB ID: 1ere). The left panels illustrate predicted binding modes of the target compounds (white sticks) at green cartoon 3D representation of the biological target. On the right, overlay of the docked compounds and crystallized ligand (E2; cyan sticks) depicting their comparative orientations within the binding site. Polar interactions (Hydrogen bonding) are illustrated as black dashed lines. Only residues (lines) located within 4 **Å** radius of bound ligand are displayed and labeled with sequence number.

Moving towards the docked plant sterol and triterpene, both stigmasterol and oleanolic acid possess comparable sesquiterpene skeletons of E2, allowing their predicted diagonal anchoring across the binding site. However, lacking the aromatic features essential for furnishing London force dispersion with Phe404, both compounds could not adopt the characteristic pincer-like arrangement. Polar interactions with Glu353 and Arg394, yet not His524, were depicted within stigmasterol and oleanolic acid poses contributing to ligand/target binding (Figure 8a,b). Nevertheless, both large sized ligands possess highly steric features, including triterpenoid fifth fused ring or 17β-branched aliphatic side-chain, directed inwards the hydrophobic subpocket at the β-face of E2’s ring-C. These features impose great steric hinderance, forcing the ligands to adopt unfavored high energy conformation (E_Conf = 265.075 and 231.779 Kcal mol^−1^) which was, by far, noncompensated via binding energy. This may account for the predicted significantly poor docking score, *S* = −1.029 and −1.012 Kcal·mol^−1^ for stigmasterol and oleanolic acid, respectively. Finally, the alkyl-phenylketone; xanthoxyline provides a unique binding pose owing to its size being much smaller than any of the investigated ligands. The ligand’s high ranked pose showed favored anchoring towards Arg394, rather than His524, due to possessing the E2’s ring-A aromatic/hydrogen bond pharmacophoric features (Figure 8c). Nevertheless, xanthoxyline lacked favored nonpolar contacts with the relevant hydrophobic residues of H8, H11, H12, as well as efficient occupancy of the binding site, which finally furnished intermediate predicted binding energy (*S* = −6.301 Kcal·mol^−1^).

**Figure 9:**
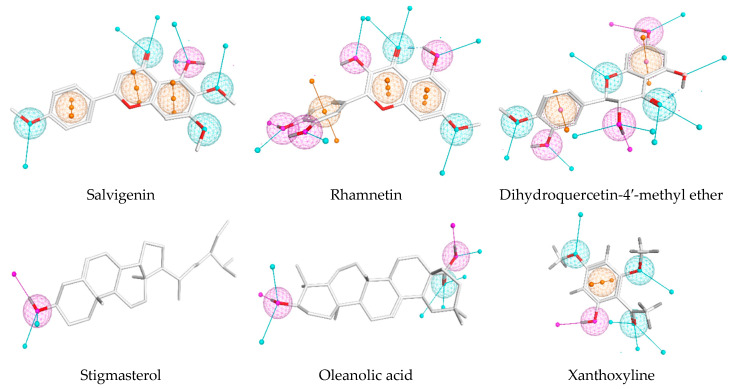
The 3D structural representation of the investigated compounds (white sticks) with their representative pharmacophoric features (mesh spheres) as well as projected virtual points (arrows). Both features and directionality of H-bond acceptor, H-bond acceptor/donor, or aromaticity are colored in cyan, purple, or orange, respectively.

Based on all above structure related binding findings, it can be concluded that sharing relevant E2’s pharmacophoric features (aromatic and terminal hydrogen bonding pattern; Figure 9), while maintaining an overall less sterically hindered molecular size, permitted preferential anchoring of the three investigated flavonoids within the *h*ER-α LBD at low energy conformations.

## 4. Conclusions

In summary, phytochemical study of a *P.* *undulata* extract afforded six compounds identified as xanthoxyline (**1**), stigmasterol (**2**), oleanolic acid (**3**) along with three methoxylated flavonoids, salvigenin (**4**), rhamnetin (**5**) and dihydroquercetin-4′-methyl ether (**6**). Both the crude extract and the above isolates (**1**–**6**) exhibited remarkable in vitro antitumour activity against a breast cancer cell line (MCF-7) and displayed promising in vivo cytotoxic effect against Ehrlich’s ascites carcinoma (EAC). Moreover, they showed significant inhibition of VEGF-B and VEGFR-2 and reduction of CD34 expression, suggesting an antiangiogenic activity through deactivation of VEGF signaling. This may account for the mechanism by which they affected tumor growth. The inhibition activity of the angiogenesis of the isolated compounds was confirmed by molecular docking simulation using *h*ER**-***α* as a potential biological target to determine the binding affinity of the isolated flavonoids towards VEGFR-2. Findings from the docking study were in good agreement with the furnished biological activity profiles, where salvigenin (**4**), rhamnetin (**5**), dihydroquercetin-4′-methyl ether (**6**), and xanthoxyline (**1**) showed the highest to intermediate docking ranks, as well as preferential binding interactions with *h*ER-α pocket’s key residues. In this regard, we propose these compounds, isolated from *P. undulata* extract, as an attractive approach to combat breast cancer via the potential inhibition of tumor angiogenesis.

## Figures and Tables

**Table 1:** IC_50_ values of the total extract and isolated compounds **1**–**6** on breast (MCF-7) cancer cell and vero lines.

Cell Line	Test Sample							
	Total Extract	1	2	3	4	5	6	Doxo *
**MCF-7**	28.10 ± 0.17	23.50 ± 0.09	47.00 ± 0.06	30.00 ± 0.14	18.00 ± 0.08	18.50 ± 0.12	22.50 ± 0.06	2.97 ± 0.06
**Vero**	>50.00	46.00 ± 0.28	>50.00	>50.00	>50.00	>50.00	>50.00	24.90 ± 0.17

Results were expressed as mean ± S.E.M of three independent experiments, significant differences was set at *p* < 0.05, (* Doxo = doxorubicin (positive control).

**Table 2:** Data of ligand docking studies on *h*ER-*α* ligand binding domain.

Ligand Name	MOE Docking Score (*S*) ^a^ (Kcal·moL^−1^)	RMSD ^b^ (**Å**)	Ligand–Target Interaction Description [Type; Length (**Å**); Angle (°); Binding Residues]
**17β-estradiol** **(Redocked) ^c^**	−7.939	0.99	H-bond; 2.1 **Å**; 136.2°; Crystallized HO**H** with 3-**O**H H-bond; 1.6 **Å**; 179.4°; Glu353-C**O**^−^ side chain with 3-O**H** H-bond; 2.2 **Å**; 144.1°; Arg394-NH**H** side chain with 3-**O**H H-bond; 2.1 **Å**; 163.2°; His524-=**N**- side chain with 17β-O**H** π-π interaction; 3.4 **Å**; Phe404 with Ring-A
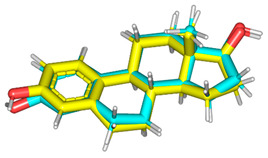			
**Salvigenin**	−11.067	1.98	H-bond; 2.0 **Å**; 128.3°; Crystallized HO**H** with p-(**O**Me)Ph H-bond; 2.4 **Å**; 131.2°; Arg394-NH**H** side chain with p-(**O**Me)Ph H-bond; 2.5 **Å**; 171.3°; His524-=**N**- side chain with 5-O**H** H-bond; 3.4 **Å**; 135.6°; Leu525-N**H** main chain with 5-OMe π-π interaction; 3.3 **Å**; Phe404 with 2-Ph
**Rhamnetin**	−15.479	1.46	H-bond; 2.3 **Å**; 132.9°; Glu353-C**O**^-^ side chain with p-(**O**H)Ph H-bond; 2.0 **Å**; 144.1°; Arg394-NHH side chain with p-(**O**H)Ph H-bond; 2.3 **Å**; 142.4°; His524-=**N**- side chain with 5-O**H** H-bond; 3.0 **Å**; 162.9°; Leu346-C=**O** main chain with m-(O**H**)Ph π-π interaction; 3.4 **Å**; Phe404 with 2-Ph
**Dihydro** **quercetin 4′-methyl ether**	−14.524	1.24	H-bond; 2.4 **Å**; 143.4°; Crystallized HO**H** with p-(**O**Me)Ph H-bond; 2.3 **Å**; 132.9°; Arg394-HN**H** side chain with p-(**O**Me)Ph H-bond; 2.0 **Å**; 163.5°; His524-=**N**- side chain with 5-O**H** π-π interaction; 3.8 **Å**; Phe404 side chain with 2-Ph
**Stigmasterol**	−1.029	1.54	H-bond; 1.7 **Å**; 156.0°; Glu353-C**O**^-^ side chain with 3β-O**H** H-bond; 2.3 **Å**; 103.3°; Arg394-HN**H** side chain with 3β-**O**H
**Oleanolic acid**	−1.012	0.93	H-bond; 2.3 **Å**; 103.4°; Arg394-HN**H** side chain with 3β-**O**H H-bond; 2.5 **Å**; 145.7°; Phe404-C=**O** main chain with 3β-OH H-bond; 2.8 **Å**; 109.4°; Gly521-C=**O** main chain with 17β-COO**H**
**Xanthoxyline**	−6.301	1.79	H-bond; 2.9 **Å**; 106.3°; Crystallized HO**H** with O**H** H-bond; 1.9 **Å**; 117.6°; Arg394-HN**H** side chain **O**H π-π interaction; 3.2 **Å**; Phe404 side chain with Ph

^a^ Scored for the best ranking pose chosen based on visual inspection, MOE (***S***) scoring function and RMSD. Refinment through rescoring by the GBVI/WSA dG scoring function. ^b^ Root-mean-square deviation (RMSD) between heavy atoms of predicted binding mode (following refinement) and those of crystallized structure (prior refinement). ^c^ Superimposing the crystallized (cyan sticks) and redocked (yellow sticks) 17*β*-estradiol ligand at *h*ER-α ligand binding domain for validating the adopted directed docking protocol.

## Data Availability

Not applicable.

## Supplementary Materials

The following are available online at https://www.mdpi.com/article/10.3390/life11080759/s1, Figure S1. Chemical structure of compound **1**; Figure S2. ^1^H-NMR spectrum of compound **1** (CDCl_3_); Figure S2-(a). Partial expansion of ^1^H-NMR spectrum of compound **1** (CDCl_3_); Figure S3. ^13^C-NMR spectrum of compound **1** (CDCl_3_); Figure S4. Chemical structure of compound **2**; Figure S5. ^1^H-NMR spectrum of compound **2** (CDCl_3_); Figure S5-(a). Partial expansion of ^1^H-NMR spectrum of compound 2 (CDCl_3_); Figure S6. ^13^C-NMR spectrum of compound **2** (CDCl_3_); Figure S7. Chemical structure of compound **3**; Figure S8. ^1^H-NMR spectrum of compound **3** (CDCl_3_); Figure S9. ^13^C-NMR spectrum of compound **3** (CDCl_3_); Figure S10. Chemical structure of compound **4**; Figure S11. ^1^H-NMR spectrum of compound **4** (CDCl_3_); Figure S11-(a,b). Partial expansions of ^1^H-NMR spectrum of compound **4** (CDCl_3_); Figure S12. ^13^C-NMR spectrum of compound **4** (CDCl_3_); Figure S12-(a,b). Partial expansions ^13^C-NMR spectrum of compound **4** (CDCl_3_); Figure S13. Chemical structure of compound **5**; Figure S14. ^1^H-NMR spectrum of compound **5** (DMSO-*d6*); Figure S15. ^13^C-NMR spectrum of compound **5** (DMSO-*d6*); Figure S16. Chemical structure of compound **6**; Figure S17. ^1^H-NMR spectrum of compound **6** (CD_3_OD); Figure S17-(a,b). Partial expansions ^1^H-NMR spectrum of compound **6** (CD_3_OD); Figure S18. ^13^C-NMR spectrum of compound **6** (CD_3_OD); Figure S18-(a,b). Partial expansions ^13^C-NMR spectrum of compound **6** (CD_3_OD).
