# Supplementary material for: VEGFR-Mediated Cytotoxic Activity of Pulicaria undulata Isolated Metabolites: A Biological Evaluation and In Silico Study
Source: Life (Basel). 2021 Jul 28;11(8):759. doi: 10.3390/life11080759 (PMC8398779; doi:10.3390/life11080759)
Supplement: Supplementary file 1 [file life-11-00759-s001.zip › life-1240393-supplementary.pdf]

VEGFR-Mediated Cytotoxic Activity of *Pulicaria undulata* Isolated Metabolites: A Biological Evaluation and In Silico Study

Sameh S. Elhady [1,†], Reda F. A. Abdelhameed [2,†], Salwa H. Zekry [2,3], Amany K. Ibrahim [2], Eman S. Habib [2], Khaled M. Darwish [4], Reem M. Hazem [5], Khadijah A. Mohammad [6], Hashim A. Hassanean [2], and Safwat A. Ahmed [2,*]

[1] Department of Natural Products, Faculty of Pharmacy, King Abdulaziz University, Jeddah 21589, Saudi Arabia; ssahmed@kau.edu.sa
[2] Department of Pharmacognosy, Faculty of Pharmacy, Suez Canal University, 41522 Ismailia, Egypt; omarreda_70@yahoo.com (R.F.A.A.); solayossif@gmail.com (S.H.Z.); am_kamal66@yahoo.com (A.K.I.); emy_197@hotmail.com (E.S.H.); hashem_omar@pharm.suez.edu.eg (H.A.H.)
[3] Department of Pharmacognosy, Faculty of Pharmacy, Sinai University, El-Arish North Sinai 45511, Egypt;
[4] Department of Medicinal Chemistry, Faculty of Pharmacy, Suez Canal University, 41522 Ismailia, Egypt; khaled_darwish@pharm.suez.edu.eg
[5] Department of Pharmacology and Toxicology, Faculty of Pharmacy, Suez Canal University, 41522 Ismailia, Egypt; reem_ahmed@pharm.suez.edu.eg
[6] Department of Pharmaceutical Chemistry, Faculty of Pharmacy, King Abdulaziz University, Jeddah 21589, Saudi Arabia; kmohammad@kau.edu.sa
* Correspondence: safwat_aa@yahoo.com or safwat_ahmed@pharm.suez.edu.eg; Tel.: (+20) 010-92638387; Fax: (+20) 064-3230741
† These authors equally contributed to this work

Additional Experimental Detail

1) **Figure S1.** Chemical structure of compound **1**
2) **Figure S2.** ^{1}H-NMR spectrum of compound **1** ($CDCl_3$, 300 MHz)
3) **Figure S2-(a).** Partial expansion of ^{1}H-NMR spectrum of compound **1** ($CDCl_3$, 300 MHz)
4) **Figure S3.** ^{13}C-NMR spectrum of compound **1** ($CDCl_3$, 100 MHz)
5) **Figure S4.** Chemical structure of compound **2**
6) **Figure S5.** ^{1}H-NMR spectrum of compound **2** ($CDCl_3$, 300 MHz)
7) **Figure S5-(a).** Partial expansion of ^{1}H-NMR spectrum of compound **2** ($CDCl_3$, 300 MHz)
8) **Figure S6.** ^{13}C-NMR spectrum of compound **2** ($CDCl_3$, 100 MHz)
9) **Figure S7.** Chemical structure of compound **3**
10) **Figure S8.** ^{1}H-NMR spectrum of compound **3** ($CDCl_3$, 300 MHz)
11) **Figure S9.** ^{13}C-NMR spectrum of compound **3** ($CDCl_3$, 100 MHz)
12) **Figure S10.** Chemical structure of compound **4**
13) **Figure S11.** ^{1}H-NMR spectrum of compound **4** ($CDCl_3$, 300 MHz)
14) **Figure S11-(a,b).** Partial expansions of ^{1}H-NMR spectrum of compound **4** ($CDCl_3$, 300 MHz)
15) **Figure S12.** ^{13}C-NMR spectrum of compound **4** ($CDCl_3$, 100 MHz)
16) **Figure S12-(a,b).** Partial expansions ^{13}C-NMR spectrum of compound **4** ($CDCl_3$, 100 MHz)
17) **Figure S13.** Chemical structure of compound **5**
18) **Figure S14.** ^{1}H-NMR spectrum of compound **5** (DMSO-d_6, 300 MHz)
19) **Figure S15.** ^{13}C-NMR spectrum of compound **5** (DMSO-d_6, 100 MHz)
20) **Figure S16.** Chemical structure of compound **6**
21) **Figure S17.** ^{1}H-NMR spectrum of compound **6** (CD_3OD, 500 MHz)
22) **Figure S17-(a,b).** Partial expansions ^{1}H-NMR spectrum of compound **6** (CD_3OD, 500 MHz)

23) **Figure S18.** ^{13}C-NMR spectrum of compound **6** (CD_3OD, 100 MHz)
24) **Figure S18-(a,b).** Partial expansions ^{13}C-NMR spectrum of compound **6** (CD_3OD, 100 MHz)

Figure S1: Chemical structure of compound **1**

Compound (1): $C_{10}H_{12}O_4$, White substance (60 mg), R_f = 0.25 (ETOAc/*n*-hexane 4:96); ^{1}H NMR (300 MHz, CD_3Cl); δ_H 6.05 (1H, d, *J*= 2.4 Hz, H-3), 5.91 (1H, d, *J*= 2.4 Hz, H-5), 3.81 (3H, s, OCH_3), 3.84 (3H, s, OCH_3), 2.60 (3H, s, CH_3), 14 (OH); ^{13}C NMR (100 MHz, CD_3Cl); δ_C 105.9 (C-1), 167.5 (C-2), 93.4 (C-3), 166.0 (C-4), 90.6 (C-5), 162.8 (C-6), 55.4 (2-OCH_3), 32.8 (CH_3), 203 (C=O). It was identified as **Xanthoxyline (2-hydroxy-4, 6-dimethoxyacetophenone)** by comparison with physical and spectral data from the literature.

1) Liu, L.; Yang, J.; Shi, Y. J. C.; biodiversity, Phytochemicals and biological activities of Pulicaria species. **2010,** 7, (2), 327-349. https://doi.org/10.1002/cbdv.200900014

2) Soares, M.G.; Felippe, A.P.V.d.; Guimarães, E.F.; Kato, M.J.; Ellena, J.; Doriguetto, A.C. 2-Hydroxy-4,6-dimethoxyacetophenone from leaves of Peperomia glabella J. Braz. Chem. Soc. **2006**, 17, 1205-1210, http://dx.doi.org/10.1590/S0103-50532006000700002.

3) Kouno, I.; Saishoji, T.; Sugiyama, M.; Kawano, N. A xylosylglucoside of xanthoxylin from sapium sebiferum root bark. Phytochemistry 1983, 22, 790-791, https://doi.org/10.1016/S0031-9422(00)86992-7

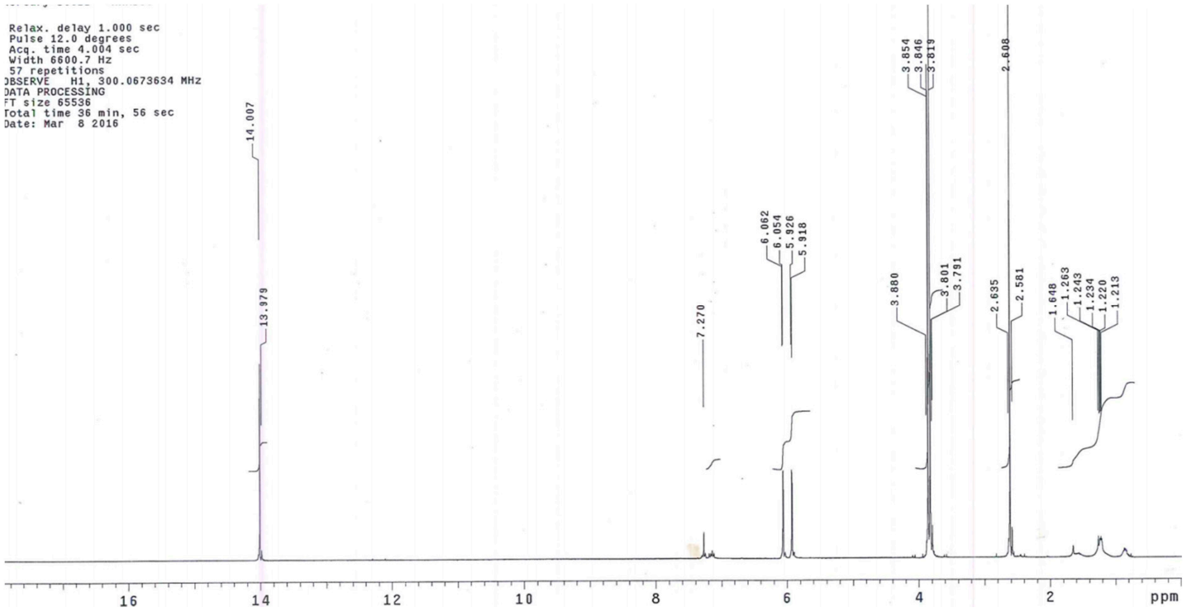

Figure S2. ^{1}H-NMR spectrum of compound **1** ($CDCl_3$, 300 MHz)

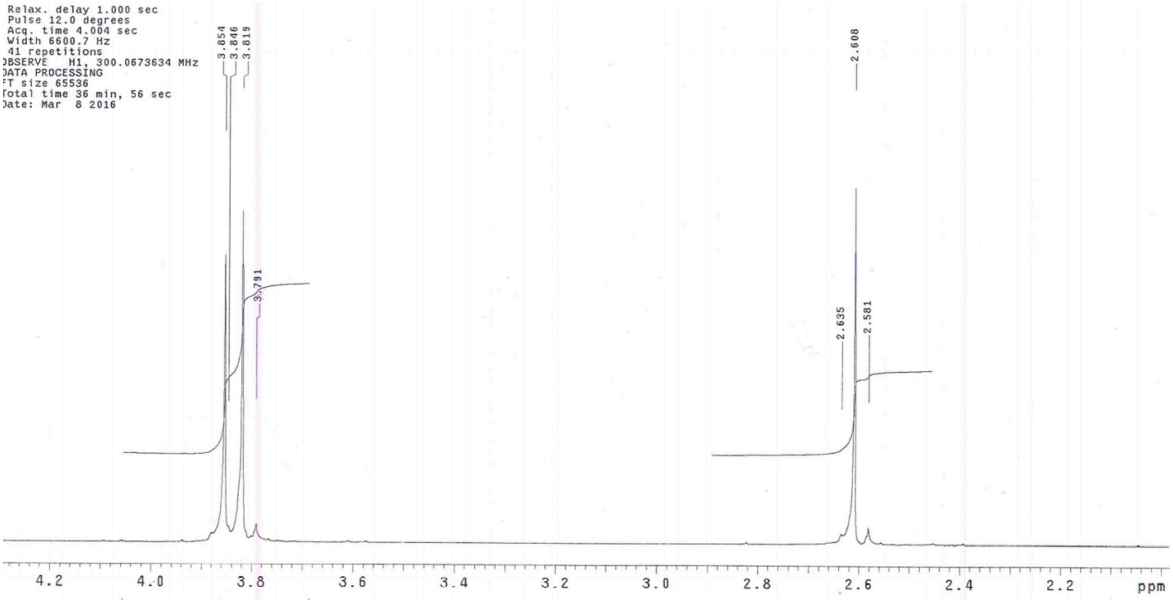

Figure S2-(a). Partial expansion of ^{1}H-NMR spectrum of compound **1** ($CDCl_3$, 300 MHz)

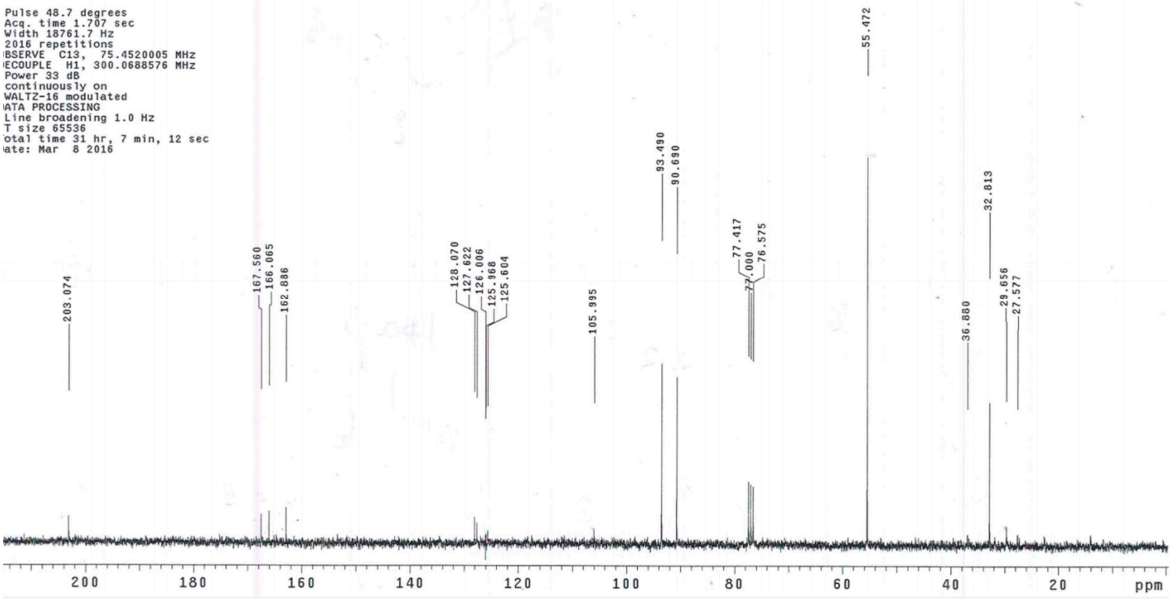

Figure S3. ^{13}C-NMR spectrum of compound **1** ($CDCl_3$, 100 MHz)

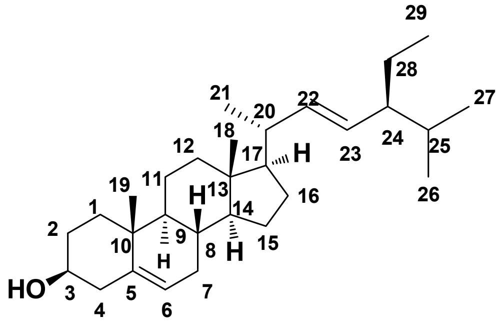

Figure S4. Chemical structure of compound **2**

Compound (2): $C_{29}H_{48}O$, White needles (90 mg), R_f = 0.23 (ETOAc/*n*-hexane 12:88); ^{1}H NMR (300 MHz, CD_3Cl); δ_H 3.51 (H-3, m), olefinic protons appeared downfield at δ_H 5.35 (H-6, m), 5.12 (H-22, m), 5.02 (H-6, m). Six methyl protons also appeared at δ_H 1.28, 1.19, 1.04, 1.00, 0.98 and 0.92 (3H each, s, CH_3). The ^{13}C-NMR has shown recognizable signals at δ_C 140.7 and 121.7, which corresponds to double bond at C-5 and C-6 double bonds respectively as well as signals at δ_C 138.2 and 129.3, which shows one more double bond in between C-22 and C-23. The δ_C value at 71.8 ppm is due to C-3 β-hydroxyl group. It was identified as **Stigmasterol** by comparison with physical and spectral data from the literature and with authentic sample.

1) Eshbakova, K.; Saidkhodzhaev, A., Triterpenoids and Sterols from Three Species of *Pulicaria*. *CHEM NAT COMPD* **2001,** 37, 196-197, https://doi.org/10.1023/A:1012307709511

2) Suttiarporn, P.; Chumpolsri, W.; Mahatheeranont, S.; Luangkamin, S.; Teepsawang, S.; Leardkamolkarn, V. Structures of Phytosterols and Triterpenoids with Potential Anti-Cancer Activity in Bran of Black Non-Glutinous Rice. *Nutrients* **2015**, *7*, https://doi.org/10.3390/nu7031672

3) Nayak, P.S.; Kar, D.M.; Nayak, S.P. ISOLATION AND CHARACTERIZATION OF STIGMASTEROL FROM CHLOROFORM FRACTION OF AERIAL PART OF ARGEMONE MEXICANA L. *International Journal of Pharmacy and Pharmaceutical Sciences* **2015**, *7*, 25-29. Online ISSN: 0975-1491, Print ISSN: 2656-0097.

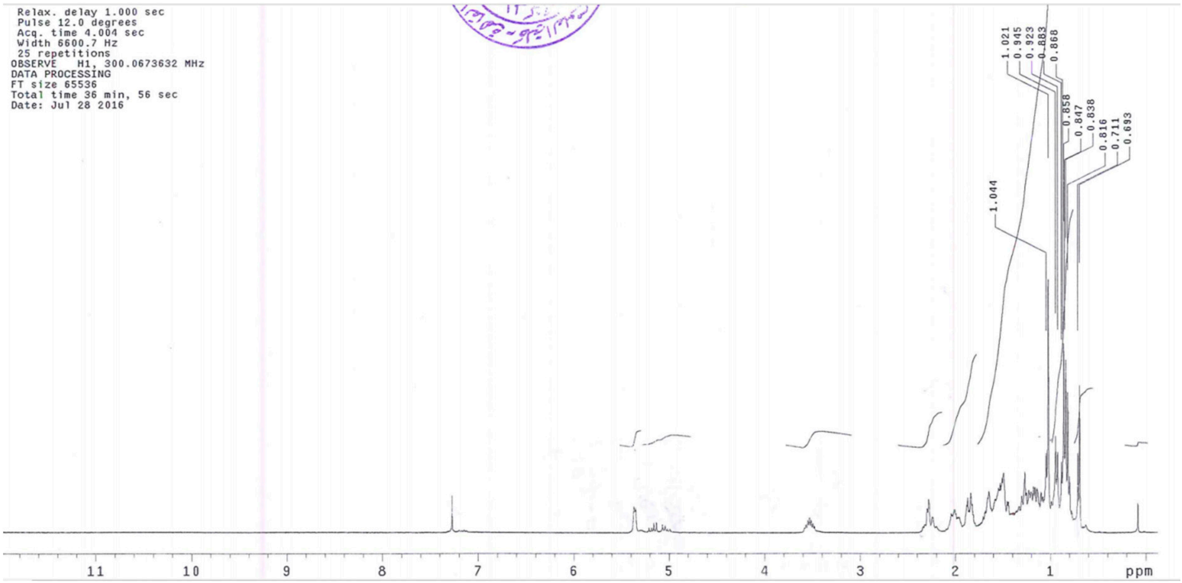

Figure S5. ^{1}H-NMR spectrum of compound **2** ($CDCl_3$, 300 MHz)

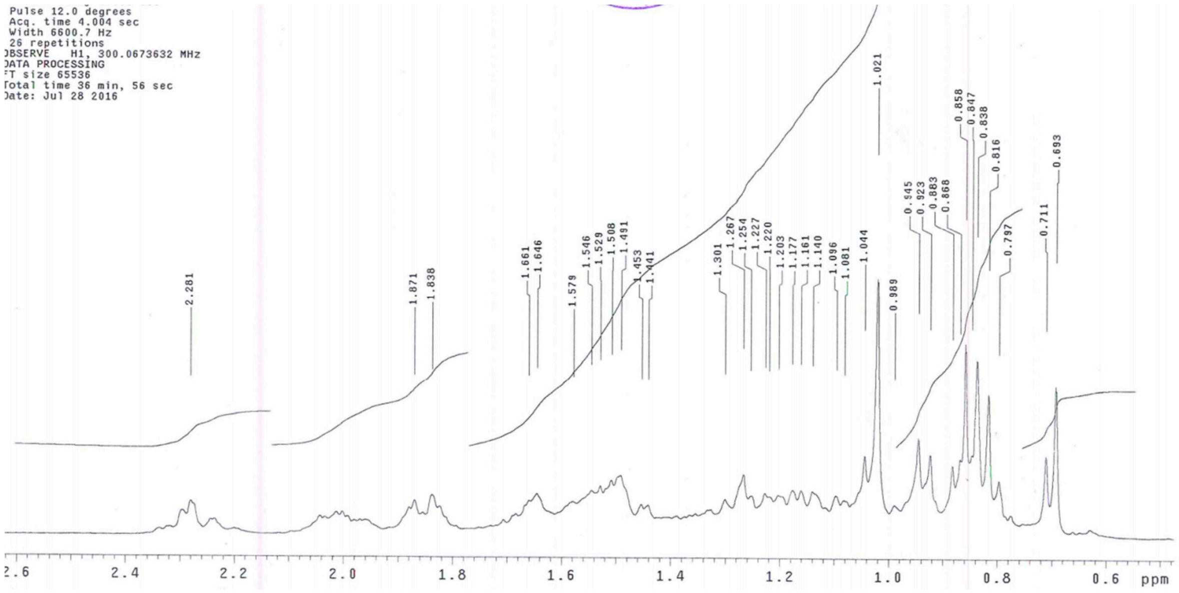

Figure S5-(a). Partial expansion of ^{1}H-NMR spectrum of compound **2** ($CDCl_3$, 300 MHz)

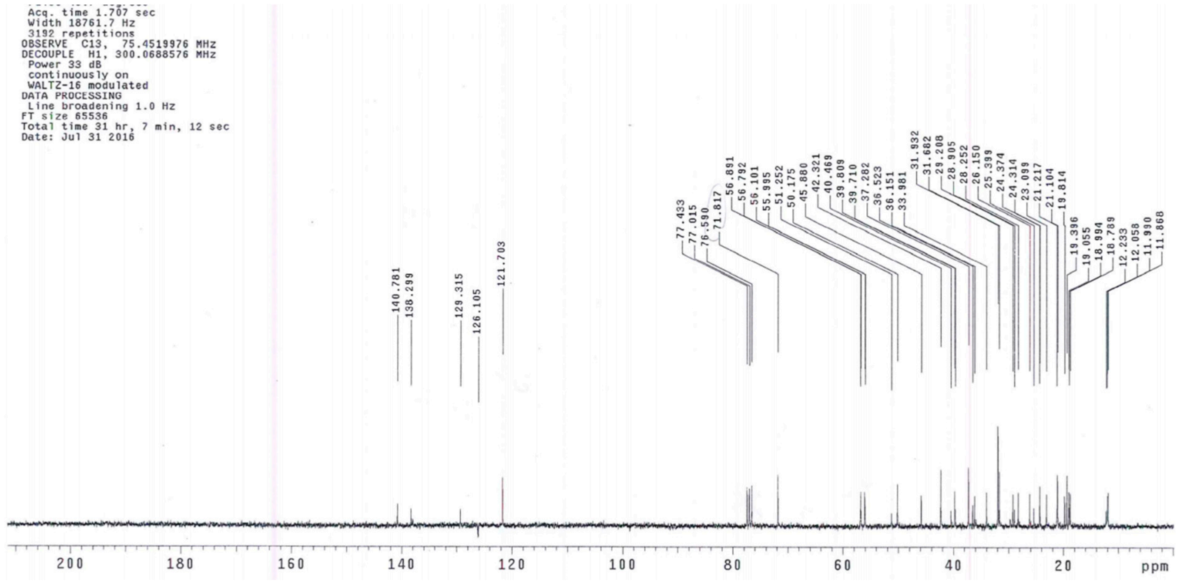

Figure S6. ^{13}C-NMR spectrum of compound **2** ($CDCl_3$, 100 MHz)

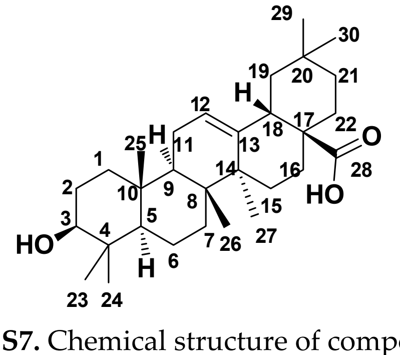

Figure S7. Chemical structure of compound **3**

Compound (3): $C_{30}H_{48}O_3$, White powder (110 mg); R_f = 0.53 (ETOAc/ *n*-hexane 30:70); ^1H NMR (300 MHz, CD_3Cl); δ_H 3.21 (H-3, m), The olefinic proton H-12, which gives a distinct characteristic triplet at δ_H 5.25 (1H, t, *J*=3.2, H-12), 2.8 (dd, *J*=13.9, 4.1, H-18). The ^{13}C NMR spectrum (100 MHz, CD_3Cl) showed a secondary hydroxyl bearing carbon at δ_C 78.7 (C-3). It also showed some recognizable signals at δ_C 55.1 (C-5), 122.1 (C-12) and 143.9 (C-13). In addition, the quaternary carbon atom (C-28) at δ_C 180.1. It was identified as **Oleanolic acid** by comparison with physical and spectral data from the literature and with authentic samples.

1) Dais, P.; Plessel, R.; Williamson, K.; Hatzakis, E., Complete ^1H and ^{13}C NMR assignment and ^{31}P NMR determination of pentacyclic triterpenic acids. *Anal. Methods* **2017,** 9, (6), 949-957. https://doi.org/10.1039/C6AY02565J

2) Hossain, M.A.; Ismail, Z. Isolation and characterization of triterpenes from the leaves of Orthosiphon stamineus. Arabian Journal of Chemistry 2013, 6, 295-298, https://doi.org/10.1016/j.arabjc.2010.10.009

3) Palu, D.; Bighelli, A.; Casanova, J.; Paoli, M. Identification and Quantitation of Ursolic and Oleanolic Acids in Ilex aquifolium L. Leaf Extracts Using (13)C and (1)H-NMR Spectroscopy. Molecules 2019, 24, https://doi.org/10.3390/molecules24234413

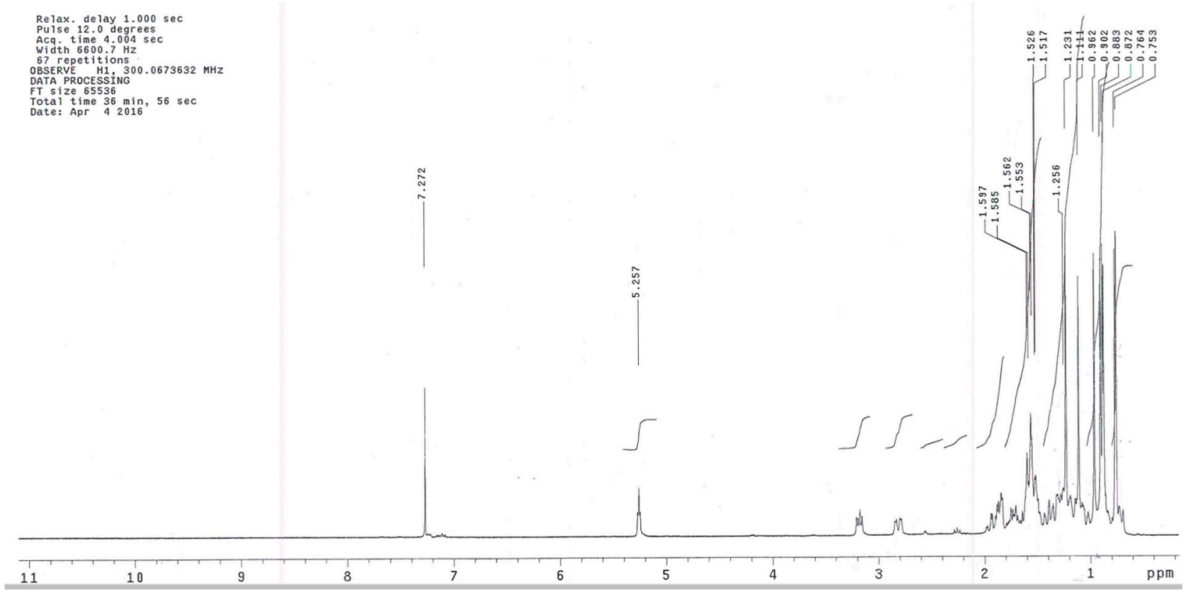

Figure S8. ^1H-NMR spectrum of compound **3** ($CDCl_3$, 300 MHz)

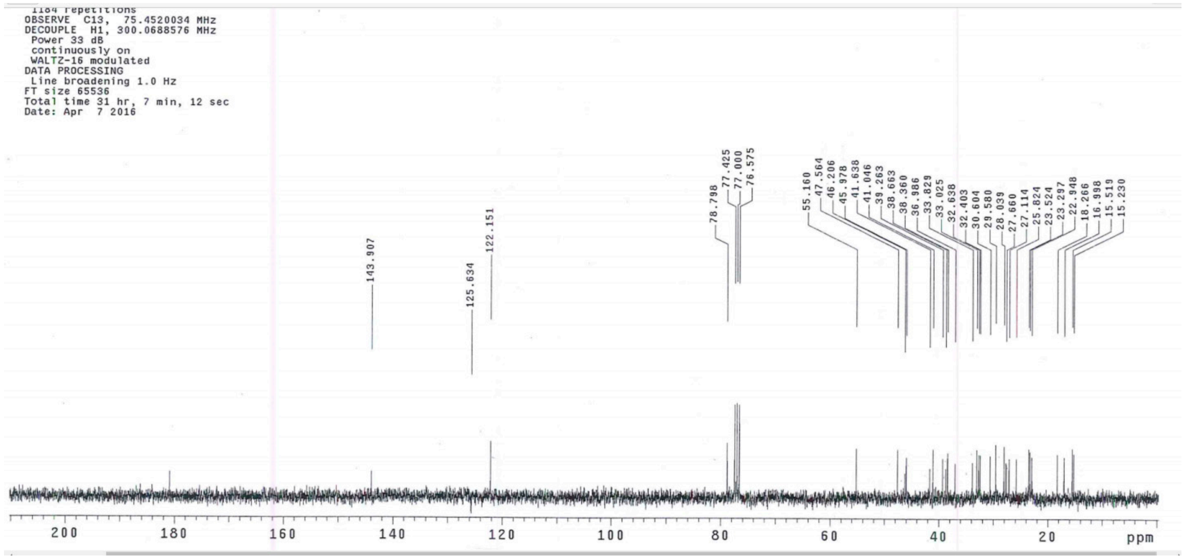

Figure S9. ^{13}C-NMR spectrum of compound **3** ($CDCl_3$, 100 MHz)

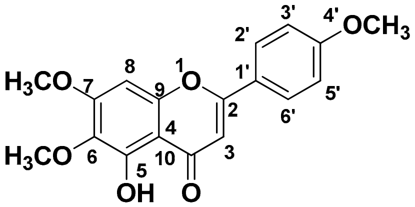

Figure S10. Chemical structure of compound **4**

Compound (4): $C_{18}H_{16}O_6$, yellow amorphous powder (55 mg); R_f = 0.36 (40:60 ETOAc/ *n*-hexane); ^{1}H NMR (300 MHz, CD_3Cl); δ_H 12.7 (1H,s, OH), 3.89 (3H, s, OCH_3), 3.92 (3H, s, OCH_3), 3.96 (3H,s, OCH_3), 6.53 (1H, s, H-3), 6.56 (1H, s, H-8), 7.02 (1H, d, *J*=2.1, H-9), 7.84 (1H, d, *J*=1.8, H-10), 7.81 (2H, d, *J*=2.1, H-2′,6′), 6.99 (2H, d, *J*=2.1 H-3′, 5′); ^{13}C NMR (100 MHz, CD_3Cl) δ_C**;** 55.5 (OCH_3), 56.2 (OCH_3), 60.7(OCH_3), 163.9 (C-2), 104 (C-3), 182.6 (C-4), 106.6 (C-5), 153.05 (C-6), 132.3 (C-7), 158.6 (C-8), 153.1 (C-9), 90.5 (C-10), 123.5C-1′, 127.9(2′,6′), 114.4 (3′,5′), 162.5 (C-4′). The ^{1}H and ^{13}C NMR data confirming the structure to be a trimethoxy substituted flavone having an additional hydroxyl group. It was identified as 5-hydroxy-4′,6,7-trimethoxyflavone **(Salvigenin)** by comparison with physical and spectral data from the literature.

1) Noori, S.; Hassan, Z. M.; Yaghmaei, B.; Dolatkhah, M., Antitumor and immunomodulatory effects of salvigenin on tumor bearing mice. *Cellular Immunology* **2013,** 286, (1), 16-21., https://doi.org/10.1016/j.cellimm.2013.10.005

2) Chaturvedula, V.S.P.; Prakash, I. Flavonoids from Astragalus propinquus. J Chem Pharm Res 2013, 5, 261-265.

3) Sen, A.; Ozbas Turan, S.; Bitis, L. Bioactivity-guided isolation of anti-proliferative compounds from endemic Centaurea kilaea. *Pharm. Biol.* **2017**, *55*, 541-546, https://doi.org/10.1080/13880209.2016.1255980

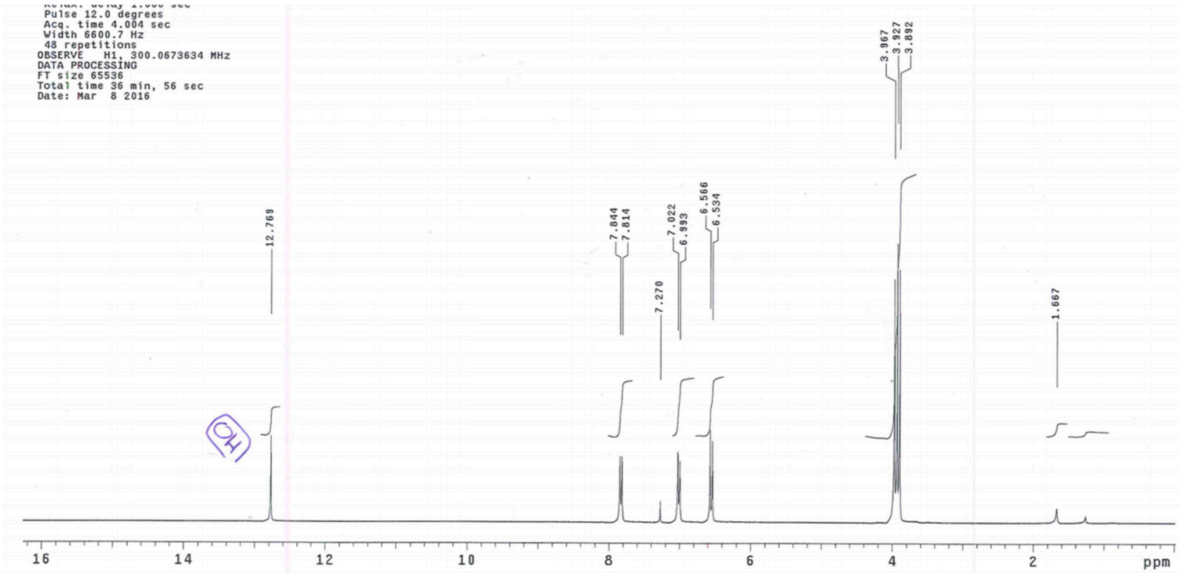

Figure S11. ^{1}H-NMR spectrum of compound **4** ($CDCl_3$, 300 MHz)

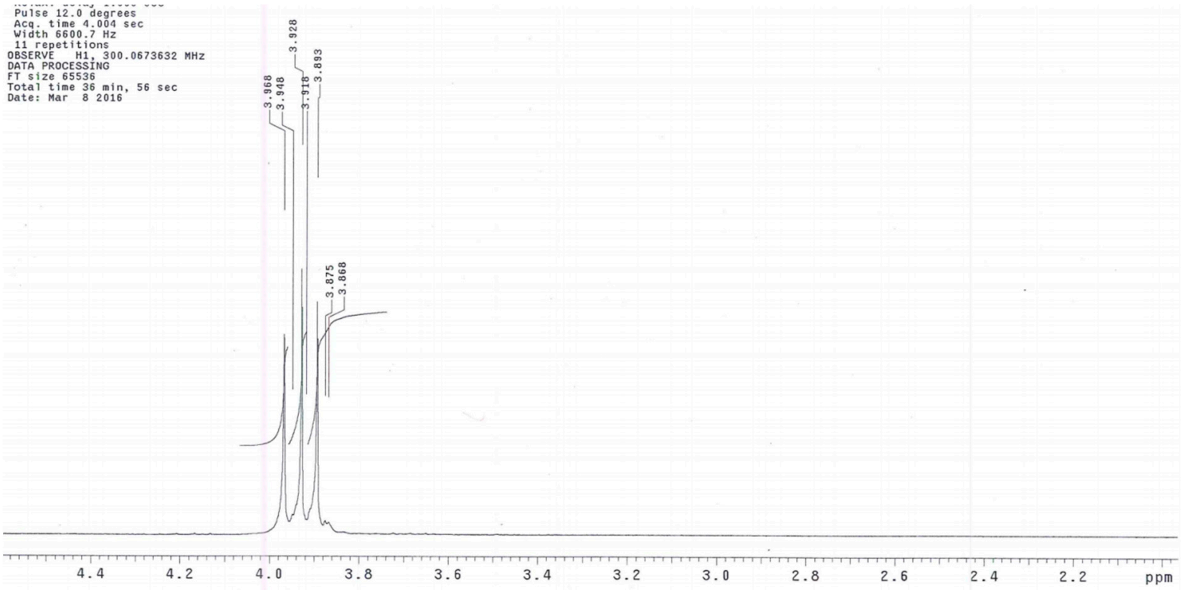

Figure S11-(a). Partial expansion of ^{1}H-NMR spectrum of compound **4** ($CDCl_3$, 300 MHz)

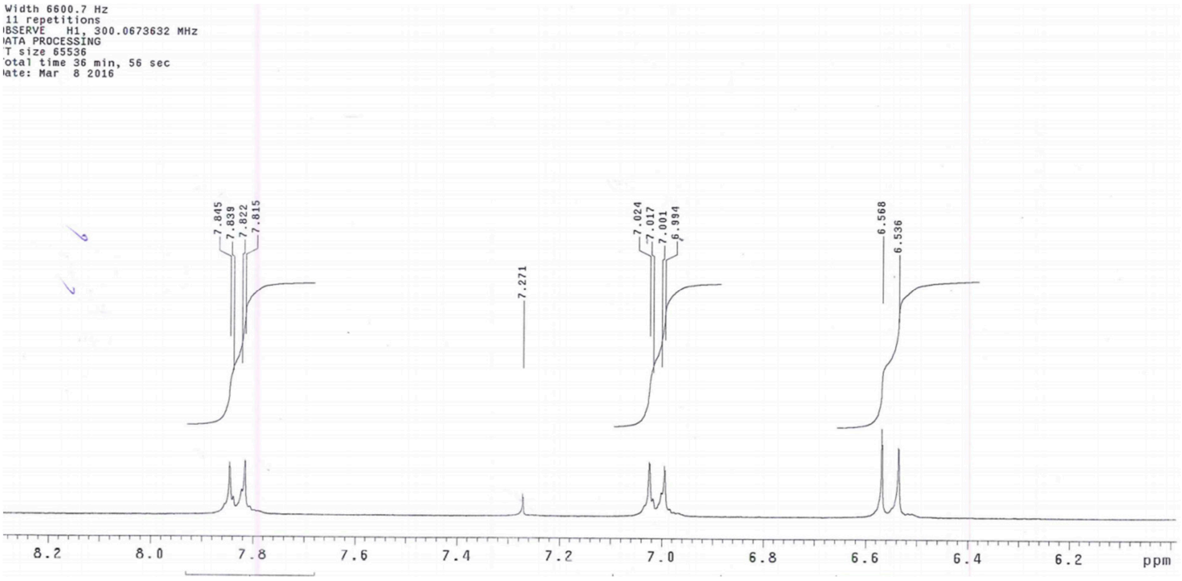

Figure S11-(b). Partial expansion of ^{1}H-NMR spectrum of compound **4** ($CDCl_3$, 300 MHz)

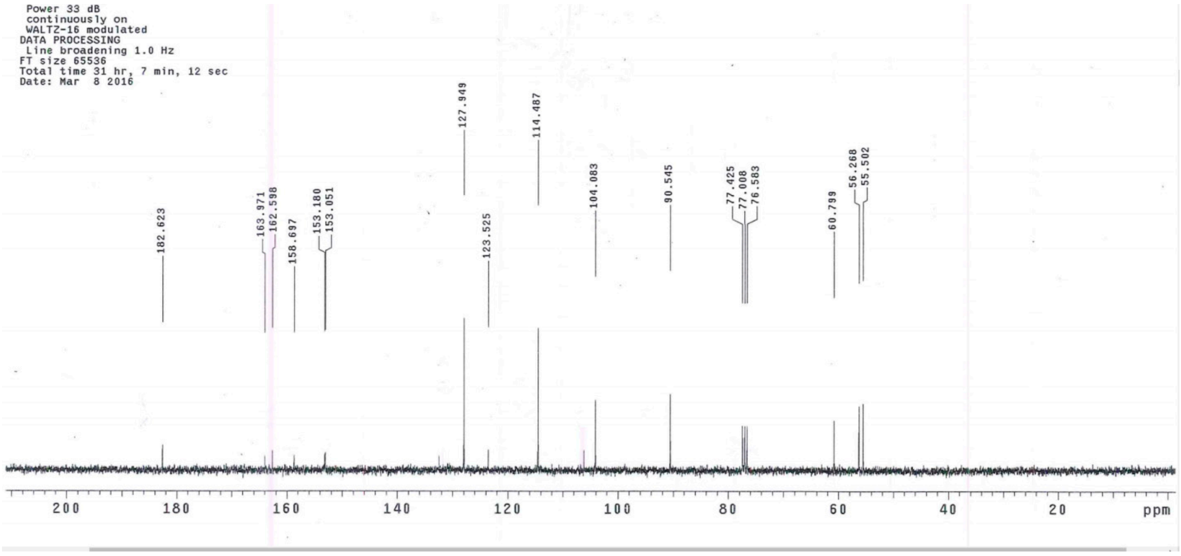

Figure S12. ^{13}C-NMR spectrum of compound **4** ($CDCl_3$, 100 MHz)

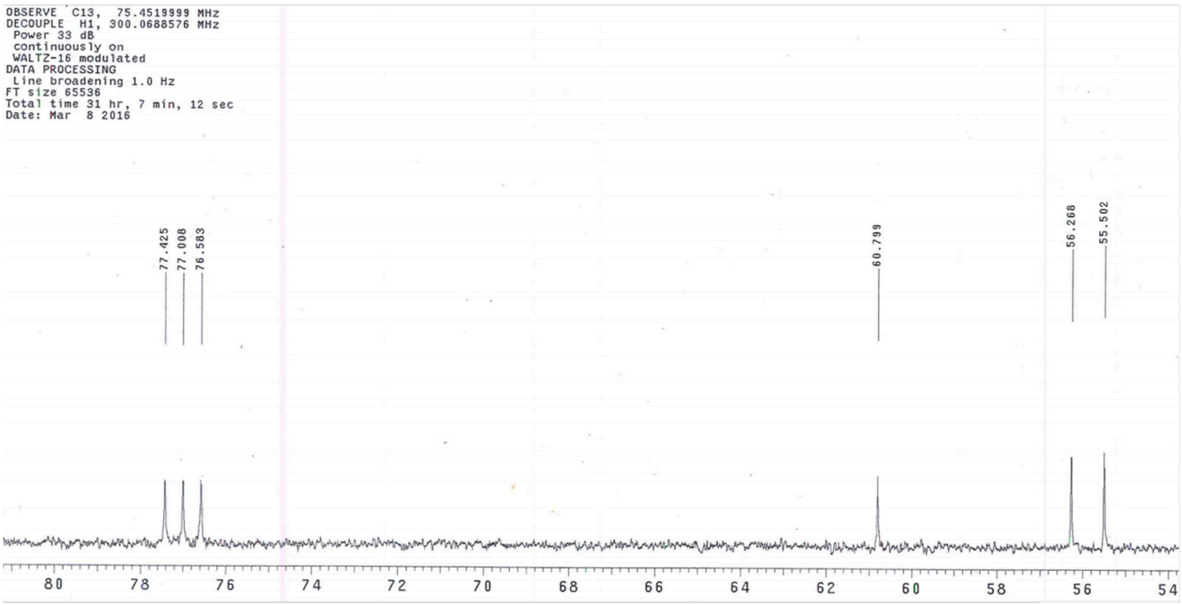

Figure S12-(a). Partial expansion ^{13}C-NMR spectrum of compound **4** ($CDCl_3$, 100 MHz)

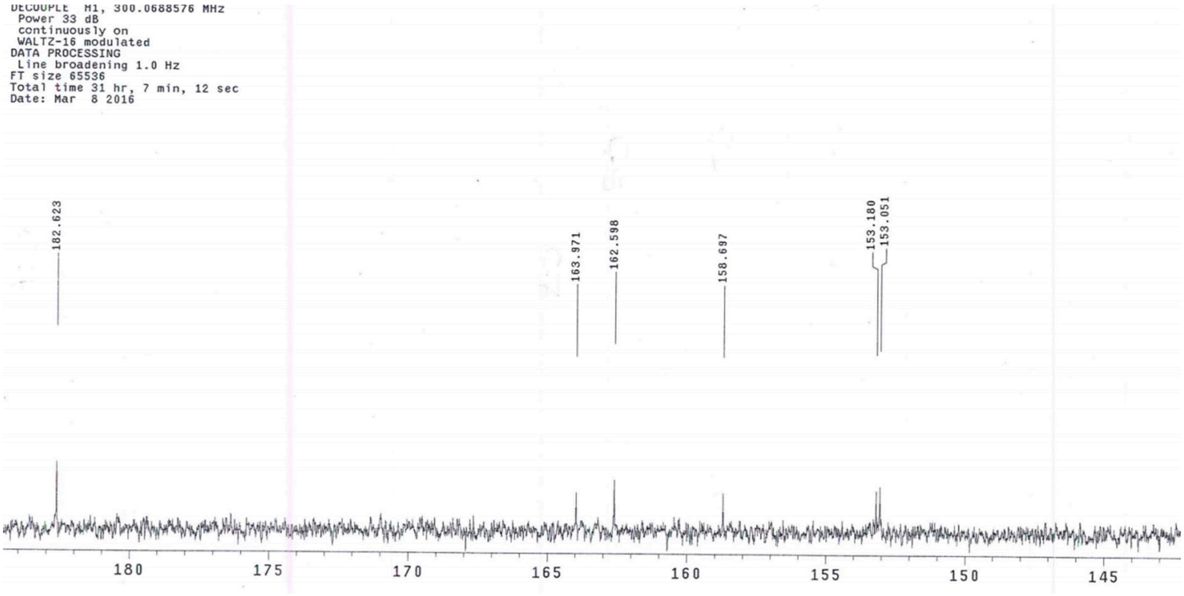

Figure S12-(b). Partial expansion ^{13}C-NMR spectrum of compound **4** ($CDCl_3$, 100 MHz)

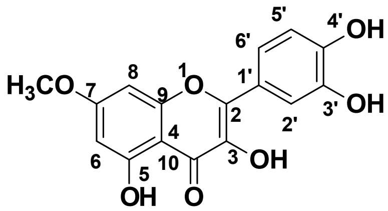

Figure S13. Chemical structure of compound **5**

Compound (5): $C_{16}H_{12}O_7$, yellow powder (70 mg); R_f = 0.27 (40:60 ETOAc/*n*-hexane); ^{1}H NMR (300 MHz, DMSO-d_6); δ_H 12.47 (1H, brs,5-OH), 9.43 (2H, brs, 4`-OH,3-OH), 7.72 (1H, d, *J*=0.6, H-2`), 7.56 (1H, dd,

J=8.1, 0.6, H-6`), 6.88 (1H, d, *J*=8.1, H-5`), 6.67 (1H, d, *J*=1.8, H-8), 6.33 (1H, d, *J*=1.8, H-6), 3.85 (OCH3); ^{13}C NMR (100 MHz, DMSO-d_6); δ_C 147.2 (C-2), 135.9 (C-3), 175.8 (C-4), 160.3 (C-5), 97.3 (C-6), 164.8 (C-7), 91.8 (C-8), 155.9 (C-9), 103.9 (C-10), 121.8 (C-1`), 115.1 (C-2`), 145 (C-3`), 147.7 (C-4`), 115.5 (C-5`), 119.9 (C-6`), (55.9 (OCH_3); It was identified as 5,3',4'-trihydroxy-7 methoxyflavonol (**Rhamnetin**) by comparison with physical and spectral data from the published literature

1) Hussein, S. R.; Marzouk, M. M.; Soltan, M. M.; Ahmed, E. K.; Said, M. M.; Hamed, A. R., Phenolic constituents of Pulicaria undulata (L.) C.A. Mey. sub sp. undulata (Asteraceae): Antioxidant protective effects and chemosystematic significances. *Journal of Food and Drug Analysis* **2017,** 25, (2), 333-339, https://doi.org/10.1016/j.jfda.2016.09.008

2) Saewan, N.; Koysomboon, S.; Chantrapromma, K. Anti-tyrosinase and anti-cancer activities of flavonoids from Blumea balsamifera DC. *J Med Plants Res* **2010**, *5*.

3) Chauhan, D.; Chauhan, J.S. Flavonoid Glycosides from Pongamia pinnata. *Pharm. Biol.* **2002**, *40*, 171-174, doi:10.1076/phbi.40.3.171.5833.

4) Lee, E.-J.; Moon, B.-H.; Park, Y.; Hong, S.-W.; Lee, S.-H.; Lee, Y.-G.; Lim, Y.-H. Effects of hydroxy and methoxy substituents on NMR data in flavonols. Bull. Korean Chem. Soc. 2008, 29, 507-510.

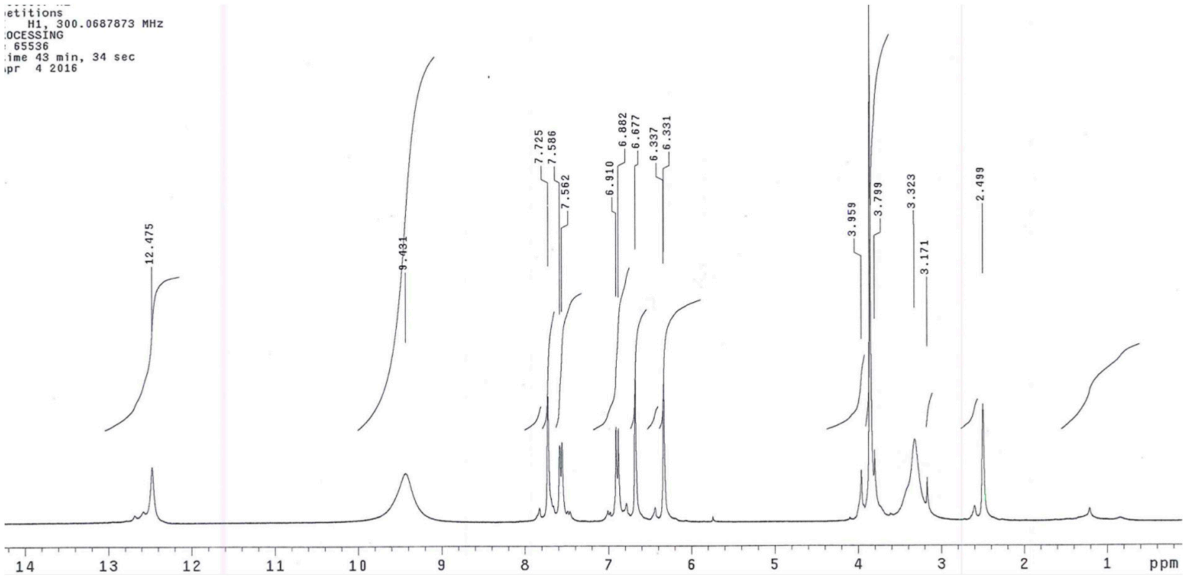

Figure S14. ^{1}H-NMR spectrum of compound **5** (DMSO-d_6, 300 MHz)

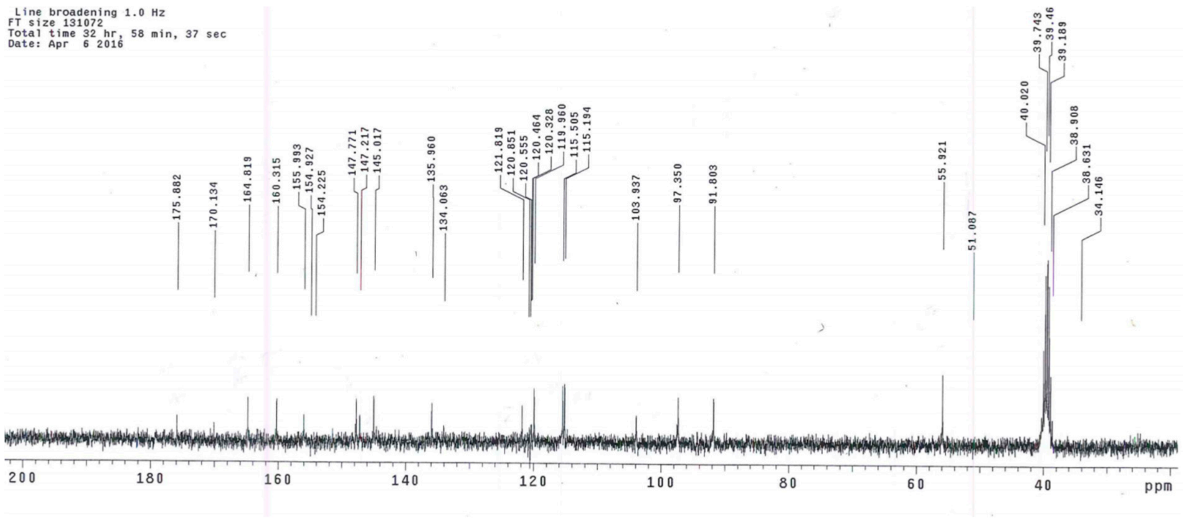

Figure S15. ^{13}C-NMR spectrum of compound **5** (DMSO-d_6, 100 MHz)

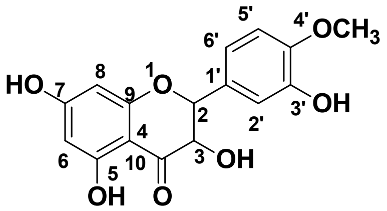

Figure S16. Chemical structure of compound **6**

Compound (6): $C_{16}H_{14}O_7$; yellow powder (65 mg); R_f = 0.46 (50:50 ETOAc/ *n*-hexane); ^{1}H NMR (500 MHz, CD_3OD); δ_H 6.94 (1H, d, *J*= 2.3, H-2'), 6.82 (1H, dd, *J* = 8.4, 2.3, H-6'), 6.77 (1H, d, *J* = 8.4, H-5'), 6.06 (1H, d, *J* = 2.3, H-8), 6.02 (1H, d, *J* = 2.3 Hz, H-6), 4.51 (1H,d, *J* = 11.45, H-3), 4.91 (1H, d, *J*= 11.45, H-2), 3.79 (3H, s, OCH_3). The ^{1}H NMR spectrum showed two protons at δ_H 4.91 and 4.51 due to H-2 and H-3 of a dihydroflavonol respectively; the trans-configuration of the dihydroflavonol unit could be deduced from the coupling constant between H-2 and H-3 (11.45 Hz). These assignments were confirmed by two oxymethine carbons appear upfield at δ_C 83.8 (C-2) and 72.3 (C-3) in the ^{13}C NMR spectrum and a typical downfield resonance characteristic of a C-4 carbonyl resonance of a flavanonol skeleton 197.5 (C-4); 163.6 (C-5), 93.6 (C-6), 168.4 (C-7), 94.6 (C-8), 162.9 (C-9), 101.2 (C-10), 128.3 (C-1′), 114.4 (C-2′), 145.7 (C-3′), 144.9 (C-4′), 115 (C-5′), 119.5 (C-6′), 55.1 (OCH3). It was identified as **Dihydroquercetin 4′-methyl ether** by comparison with physical and spectral data from the literature.

1) Lee, IC., Bae, JS., Kim, T. *et al.* Polyphenolic constituents from the aerial parts of *Thymus quinquecostatus* var. *japonica* collected on ulleung island. *J Korean Soc Appl Biol Chem* **54,** 811–816 (2011). https://doi.org/10.1007/BF03253166

2) Algabr, M. Flavonoids from Pulicaria jaubertii (Asteraceae) from Yemen. Res. j. pharm 2015.

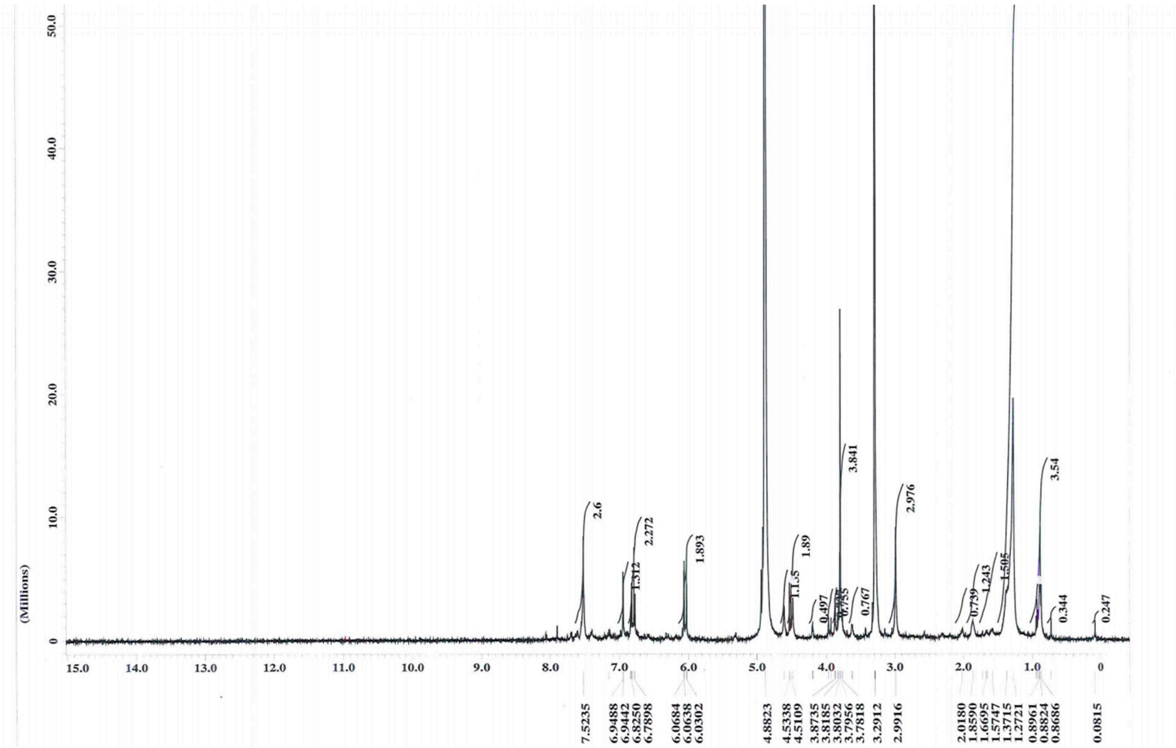

Figure S17. ^{1}H-NMR spectrum of compound **6** (CD_3OD, 500 MHz)

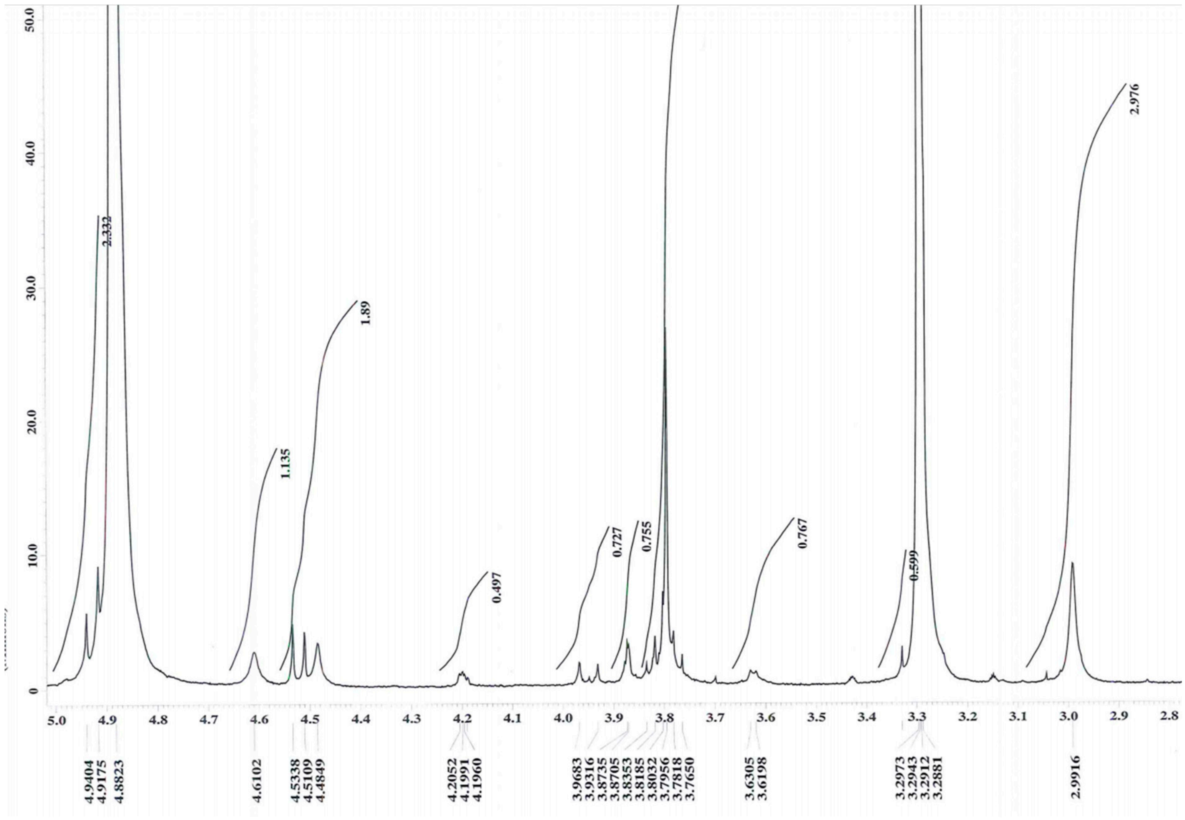

Figure S17-(a). Partial expansion ^{1}H-NMR spectrum of compound **6** (CD_3OD, 500 MHz)

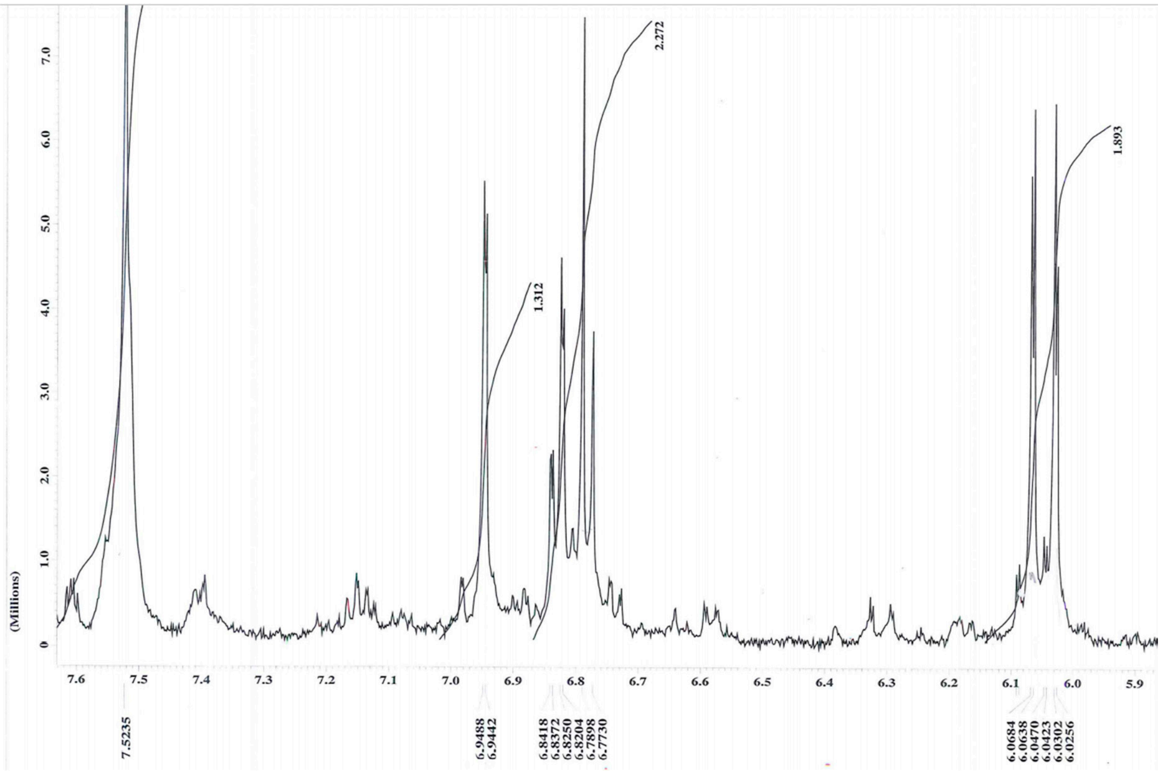

Figure S17-(b). Partial expansion ^{1}H-NMR spectrum of compound **6** (CD_3OD, 500 MHz)

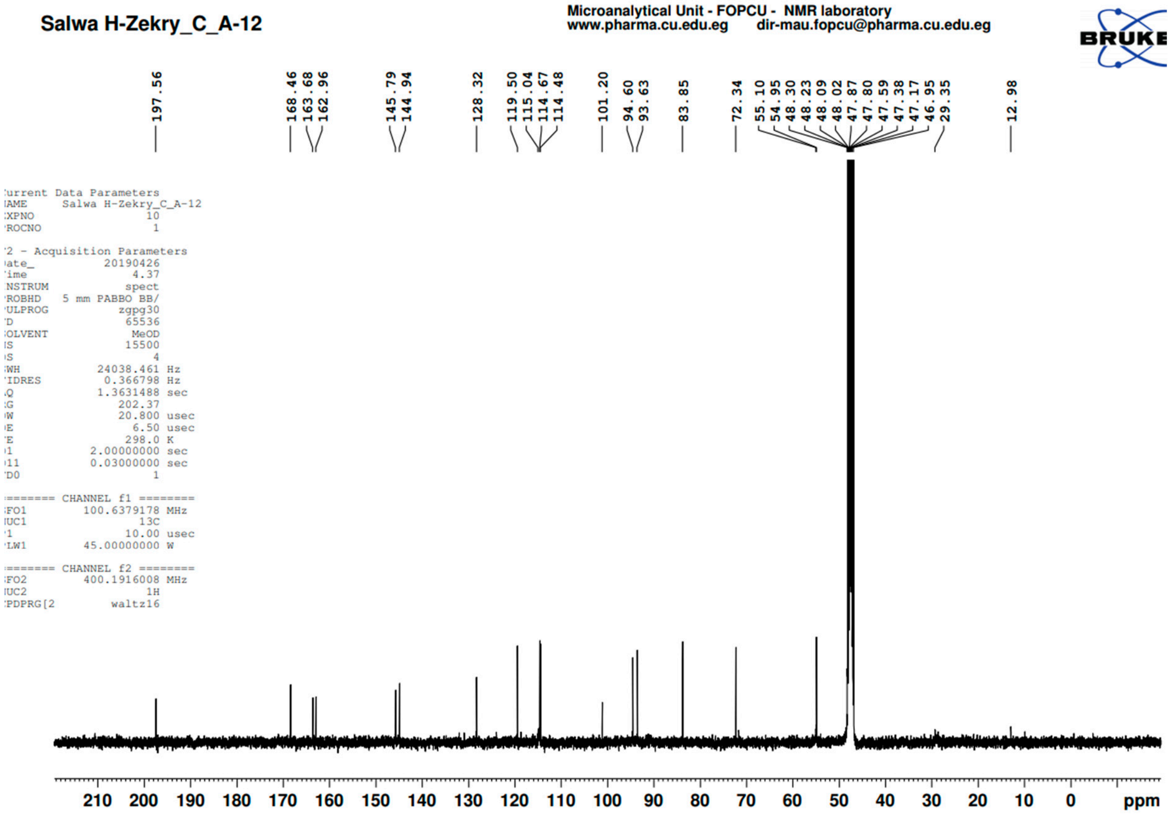

Figure S18. ^{13}C-NMR spectrum of compound **6** (CD_3OD, 100 MHz)

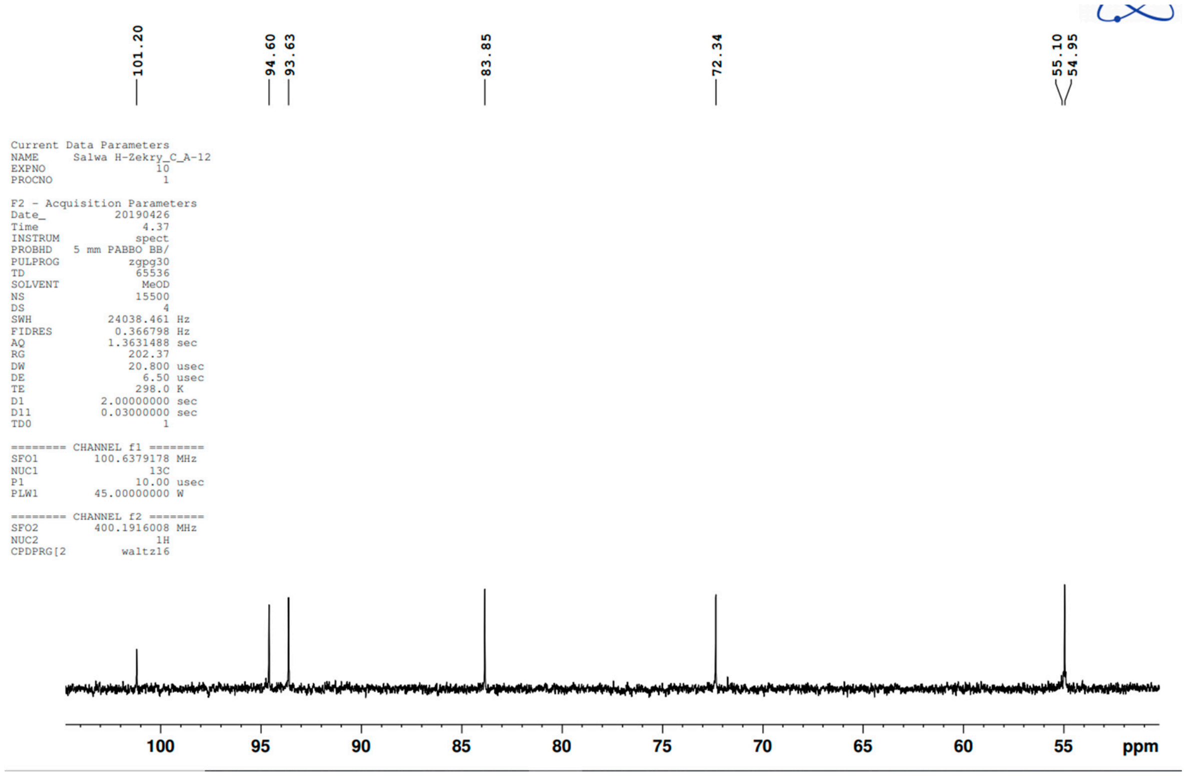

Figure S18-(a). Partial expansion ^{13}C-NMR spectrum of compound **6** (CD_3OD, 100 MHz)

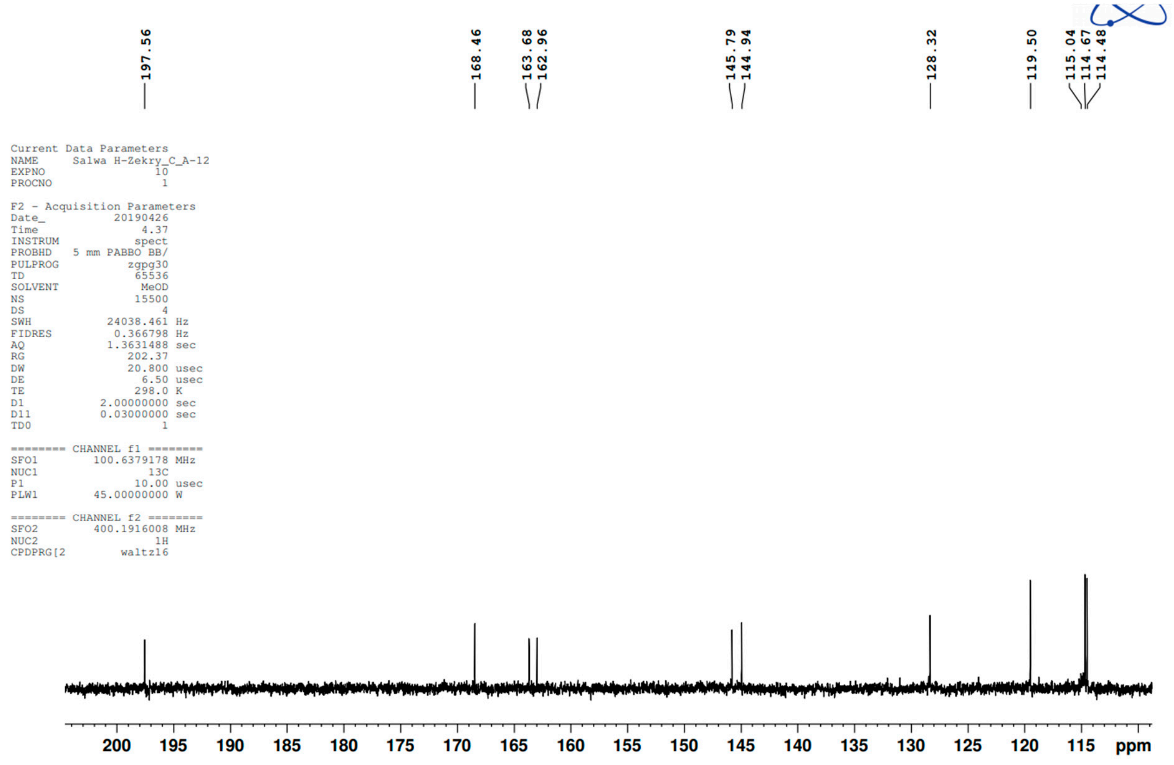

Figure S18-(b). Partial expansion ^{13}C-NMR spectrum of compound **6** (CD_3OD, 100 MHz)
